# Proteomics-based investigation of cerebrovascular molecular mechanisms in cerebral amyloid angiopathy by the FFPE-LMD-PCT-SWATH method

**DOI:** 10.1186/s12987-022-00351-x

**Published:** 2022-07-01

**Authors:** Takumi Handa, Hayate Sasaki, Masaki Takao, Mitsutoshi Tano, Yasuo Uchida

**Affiliations:** 1 Graduate School of Pharmaceutical Sciences, Tohoku University, Sendai, Japan; 2 Department of Neurology and Brain Bank, Mihara Memorial Hospital, Isesaki, Japan; 3 Department of Clinical Laboratory, National Center of Neurology and Psychiatry, National Center Hospital, Kodaira, Japan; 4 Division of Membrane Transport and Drug Targeting, Graduate School of Pharmaceutical Sciences, Tohoku University, 6-3 Aoba, Aramaki, Aoba-ku, Sendai, 980-8578 Japan

**Keywords:** CAA, Fibrosis, Aβ, Apoptosis, SWATH

## Abstract

**Background:**

Cerebral amyloid angiopathy (CAA) occurs in 80% of patients with Alzheimer’s disease (AD) and is mainly caused by the abnormal deposition of Aβ in the walls of cerebral blood vessels. Cerebrovascular molecular mechanisms in CAA were investigated by using comprehensive and accurate quantitative proteomics.

**Methods:**

Concerning the molecular mechanisms specific to CAA, formalin-fixed paraffin-embedded (FFPE) sections were prepared from patients having AD neuropathologic change (ADNC) with severe cortical Aβ vascular deposition (ADNC +/CAA +), and from patients having ADNC without vascular deposition of Aβ (ADNC +/CAA −; so called, AD). Cerebral cortical vessels were isolated from FFPE sections using laser microdissection (LMD), processed by pressure cycling technology (PCT), and applied to SWATH (sequential window acquisition of all theoretical fragment ion spectra) proteomics.

**Results:**

The protein expression levels of 17 proteins in ADNC +/CAA +/H donors (ADNC +/CAA + donors with highly abundant Aβ in capillaries) were significantly different from those in ADNC +/CAA − and ADNC −/CAA − donors. Furthermore, we identified 56 proteins showing more than a 1.5-fold difference in average expression levels between ADNC +/CAA + and ADNC −/CAA − donors, and were significantly correlated with the levels of Aβ or Collagen alpha-2(VI) chain (COL6A2) (CAA markers) in 11 donors (6 ADNC +/CAA + and 5 ADNC −/CAA −). Over 70% of the 56 proteins showed ADNC +/CAA + specific changes in protein expression. The comparative analysis with brain parenchyma showed that more than 90% of the 56 proteins were vascular-specific pathological changes. A literature-based pathway analysis showed that 42 proteins are associated with fibrosis, oxidative stress and apoptosis. This included the increased expression of Heat shock protein HSP 90-alpha, CD44 antigen and Carbonic anhydrase 1 which are inhibited by potential drugs against CAA.

**Conclusions:**

The combination of LMD-based isolation of vessels from FFPE sections, PCT-assisted sample processing and SWATH analysis (FFPE-LMD-PCT-SWATH method) revealed for the first time the changes in the expression of many proteins that are involved in fibrosis, ROS production and cell death in ADNC +/CAA +  (CAA patients) vessels. The findings reported herein would be useful for developing a better understanding of the pathology of CAA and for promoting the discovery and development of drugs and biomarkers for CAA.

**Supplementary Information:**

The online version contains supplementary material available at 10.1186/s12987-022-00351-x.

## Background

Cerebral amyloid angiopathy (CAA) occurs in 80% of patients with Alzheimer’s disease (AD) and is mainly caused by the abnormal deposition of Aβ in the walls of cerebral blood vessels. This deposition ultimately leads to fibrosis and apoptosis of vascular endothelial cells, which can lead to fatal conditions such as strokes and cerebral haemorrhages [1, 2]. However, there is still no effective therapy for this disease and, given its widespread nature, it is important to find drug targets for CAA. It is therefore important to elucidate the pathological molecular mechanisms that are operative in the cerebral vasculature of patients with CAA.

Compared to conventional shotgun proteomics, the SWATH method is a highly accurate and reproducible comprehensive proteomics method, which is very useful for quantitatively determining the levels of expression of proteins in pathological conditions [3]. Because Aβ accumulates in cortical blood vessels of CAA patients, it is necessary to selectively collect blood vessels as samples. The laser microdissection (LMD) of blood vessels in formalin-fixed, paraffin-embedded (FFPE) sections has been shown to be effective for this purpose, but the proteomics data obtained from FFPE sections are not as robust as those from unfixed fresh tissue due to the methylene cross-linking in FFPE sections. Because of this, it has been difficult to accurately clarify the pathological molecular mechanisms for this condition using FFPE sections for collecting proteomics data. However, we recently used pressure cycling technology (PCT) to decross-link samples at a high temperature and pressure (3000 times higher than atmospheric pressure) to completely eliminate cross-linking [3]. We refer to SWATH proteomics using the FFPE sections in combination of PCT-assisted sample process as the ‘FFPE-PCT-SWATH method’. By employing our established FFPE-PCT-SWATH method, we were able to obtain the proteomics data that clearly reflect the molecular mechanisms by using unfixed fresh tissue [3].

In this study, we isolated blood vessels from FFPE sections using LMD, performed the PCT-assisted sample process and SWATH analysis to elucidate the molecular mechanisms underlying CAA. In order to elucidate the molecular mechanism specific to CAA pathology, we prepared the FFPE sections not only from patients having AD neuropathologic change (ADNC) with severe vascular deposition of cortical Aβ (ADNC +/CAA + group), but also from the patients having the ADNC but with no vascular deposition of Aβ (ADNC +/CAA − group).

## Materials and methods

### Human brain FFPE sections

**Fig. 1:**
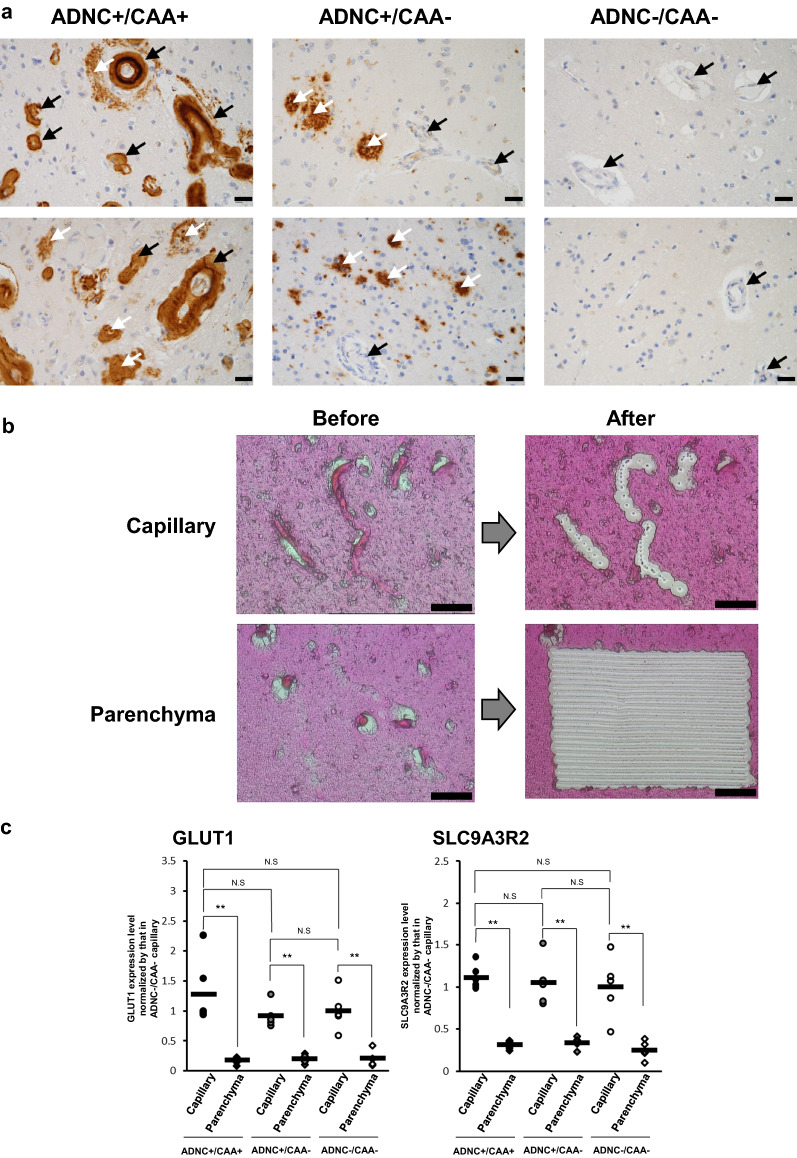
Isolation of cerebral cortical capillary and parenchyma by LMD and the validation of the purity of the isolated capillaries. **a** Aβ immunostaining was performed using the FFPE sections from ADNC +/CAA +, ADNC +/CAA −, and ADNC −/CAA − groups as previously described [5]. Two photographs in different regions are shown for each group. Black arrows indicate the cerebral vessels in occipital cortex. White arrows indicate the Aβ deposits in cortical parenchyma (except for vessels). Scale bar, 20 µm. All vessels in cortical regions were covered with Aβ in ADNC +/CAA + sections. For each group, the neighboring sections were used for the proteomic analysis (eosin staining). **b** Photographs of eosin staining before and after LMD. Scale bar, 50 µm. The eosin staining enabled to identify the cerebral vessels and cortex regions in the FFPE sections. The vessels (except for large vessels) and parenchyma in cortex were isolated by LMD as shown in the photographs until the total dissected area reaches 15 mm^2^ (× 20 μm thickness = 0.3 mm^3^) for each sample. **c** Relative protein expression levels of two endothelial cell markers in the collected capillaries and parenchyma. The expression level of each protein was normalized by the average of the protein expression level in ADNC −/CAA − capillaries as described in the "Materials and methods" section. Black plot, ADNC +/CAA +; Gray plot, ADNC +/CAA −; White plot, ADNC −/CAA −. Circle, capillary samples; Diamond, parenchymal samples. **BH-adjusted p < 0.01, significantly different between two groups. *NS* not significantly different (BH-adjusted p > 0.01)

The neuropathological information of the occipital lobes where the FFPE sections were obtained are all summarized in Table 1. The FFPE sections were provided by the Mihara Memorial Hospital brain bank. The neuropathological evaluation was based on “The NIA Alzheimer’s disease research centers program (v11)” in the National Alzheimer’s Coordinating Center (https://naccdata.org/data-collection/forms-documentation/np-11). National Institute on Aging-Alzheimer’s Association (NIA-AA) guidelines (2012) were applied to determine the level of AD pathological change [4] on the basis of a previously described neuropathological analysis [5]. Whether the AD brain includes the pathology of CAA was determined by immunohistochemical analysis for Aβ vascular deposition (Fig. 1). When the level of AD pathological changes was “intermediate to high” with moderate to severe CAA, the cases were assigned to the ADNC +/CAA + group. The Aβ depositions for this group were observed in all of the cortical vessels in FFPE sections. Cases having “intermediate” pathologic changes without CAA were assigned to the ADNC +/CAA − group. Cases having no immunoreactive deposits of Aβ were assigned to the ADNC −/CAA − group. The expression levels of Aβ and COL6A2 described in Table 1 are the experimental results obtained by the SWATH analysis in the present study (not by ELISA), and were not used for the purpose of distinguishing between the ADNC +/CAA +, ADNC +/CAA − and ADNC −/CAA − groups. The total levels of Aβ40 and Aβ42 (not separately) were quantified by the peak area of the tryptic peptide “LVFFAEDVGSNK” which is shared by Aβ40 and Aβ42. The CAA pathology of the occipital lobe was the most pronounced [6], with few neurofibrillary tangles and no synuclein deposits. For this reason, the occipital lobe was used because it is better suited than other brain regions for elucidating CAA-derived pathological molecular mechanisms. The protocols for the present study were approved by the Ethics Committees of the Mihara Memorial Hospital (protocol code 095-06, approved on January 16th 2019) and the Graduate School of Pharmaceutical Sciences, Tohoku University (protocol code 18-03, approved on December 20th 2018). Written informed consent was obtained from all subjects involved in the study.

**Table 1:** Information on the donors of the occipital lobe sections that were used in the present study

Donor number	Gender	Age	Aβ expression level normalized by average in ADNC −/CAA − capillary	COL6A2 expression level normalized by average in ADNC −/CAA − capillary	PMI (hours)	Thal phase for amyloid plaques by IHC	Braak stage for neurofibrillary degeneration	Neuritic plaque CERAD	NIA-AA Alzheimer’s disease neuropathologic change	NIA-Reagan criteria for Alzheimer's disease	CAA	Old macroinfarct	Old microinfarct (lacunar infarct)	Intracerebral hemorrhage
ADNC +/CAA +														
1 (H)	Male	90	239	1.76	5	5	4	3	2	4	3	–	+	+ , due to CAA
2 (H)	Female	93	57.9	2.41	5	3	4	2	2	2	3	–	–	Old subcortical hemorrhage frontal lobe due to CAA
3 (H)	Female	94	12.7	3.10	2	5	5	3	3	3	2	–	–	–
4 (L)	Male	69	8.12	1.13	41.5	4	5	3	3	3	3	–	–	–
5 (L)	Female	94	4.23	1.72	1	4	4	3	2	4	2	–	–	–
6 (L)	Male	84	2.07	1.31	1.5	3	3	2	2	2	2	–	–	Old subdural hematoma due to trauma
ADNC +/CAA −														
1	Female	93	4.04	1.40	8.5	3	4	3	2	4	0	–	–	Old subdural hematoma due to trauma
2	Female	111	1.97	2.31	11	3	3	2	2	2	0	–	+ , cerebral cortex and basal ganglia	–
3	Male	80	1.51	0.690	4	4	3	2	2	2	1	–	–	Thalamic hemorrhage
4	Female	90	0.807	1.00	3	4	3	2	2	2	0	Right, opposite side of the studied section	–	–
5	Female	96	0.655	1.71	7.5	4	3	2	2	2	0	–	–	–
ADNC −/CAA −														
1	Male	69	1.71	0.876	1.5	0	1	0	0	4	0	–	Lacunar infarct	–
2	Male	89	1.64	1.26	9	0	3	0	0	4	0	–	Lacunar infarct	–
3	Female	74	0.705	1.23	2.5	0	0	0	0	0	0	–	–	Cerebellar hemorrhage
4	Female	71	0.488	0.607	3.5	0	1	0	0	4	0	Cardiac embolism	–	–
5	Female	61	0.457	1.02	2.5	0	2	0	0	4	0	–	–	–

FFPE sections of occipital lobes were provided by Mihara Memorial Hospital brain bank. Neuropathological evaluation is based on “The NIA Alzheimer’s disease research centers program (v11)” in National Alzheimer’s Coordinating Center (https://naccdata.org/data-collection/forms-documentation/np-11). National Institute on Aging-Alzheimer’s Association (NIA-AA) guidelines (2012) were applied to determine the level of AD pathological change on the basis of neuropathological analysis. Thal phase for amyloid plaques by immunohistochemistry (IHC) (Thal’s phase), Phase 0–5; Braak stage for neurofibrillary degeneration (Phospho-Tau IHC by AT8 antibody), Stage I–VI; Neuritic plaque

CERAD, 0 = No neuritic plaques, 1 = Sparse neuritic plaques, 2 = Moderate neuritic plaques, 3 = Frequent neuritic plaques; NIA-AA Alzheimer’s disease neuropathologic change (ADNC), 0 = Not AD, 1 = Low ADNC, 2 = Intermediate ADNC, 3 = High ADNC; NIA-Reagan criteria for Alzheimer's disease, 0 = not AD, 1 = low likelihood, 2 = intermediate likelihood, 3 = high likelihood, 4 = unclassified. Whether the AD brain includes the pathology of CAA is determined by immunohistochemical analysis for Aβ vascular deposition. CAA, 0 = none, 1 = mild, 2 = moderate, 3 = severe. When the level of AD pathological changes was “intermediate to high” with moderate to severe CAA, the cases were assigned to ADNC +/CAA + group. The Aβ depositions for this group were observed in all the cortical vessels in FFPE sections. The cases having “intermediate” pathologic changes without CAA were assigned to ADNC +/CAA − group. The cases having no immunoreactive deposits of Aβ were assigned to ADNC −/CAA − group. CAA scores were statistically significantly larger in ADNC +/CAA + group than those in the other two groups (p < 0.001). The expression levels of Aβ and COL6A2 described in this table are the experimental results obtained by the SWATH analysis of the isolated capillary samples in the present study (not by ELISA), and were not used for the purpose to distinguish the ADNC +/CAA +, ADNC +/CAA − and ADNC −/CAA − groups. The expression levels of Aβ and COL6A2 in the capillary were normalized by those in the ADNC −/CAA − capillary as described in "Materials and methods" section. For the ADNC +/CAA + group, the three donors with highly abundant Aβ in capillaries (donors 1, 2 and 3) were classified as “ADNC +/CAA +/H”, and the other three donors (donors 4, 5 and 6) were classified as “ADNC +/CAA +/L”. The total levels of Aβ40 and Aβ42 (not separately) were quantified by the peak area of the tryptic peptide “LVFFAEDVGSNK” which is shared in Aβ40 and Aβ42. The expression levels of Aβ and COL6A2 were not statistically significantly different among three groups (p > 0.05), except for the comparison of ADNC +/CAA + and ADNC −/CAA − group for COL6A2 (p < 0.05). PMI (hours), post mortem interval

### Preparation of FFPE sections for proteomics analysis

Sections (thickness of 20 μm) were obtained from FFPE blocks, and the sections were mounted on a director lazer microdissection slide (AMR Inc., Cat. 50001-024, Tokyo, Japan). Slide-mounted FFPE sections were incubated in xylene (3 × 5 min), then absolute ethanol (2 × 1 min), 95% (v/v) ethanol (2 × 1 min), 70% ethanol (2 × 1 min), and Milli-Q water (1 min). Deparaffinized sections were incubated in Milli-Q water (5 min), stained with eosin (1 min), and then washed in running water (15 min).

### Laser microdissection (LMD)

The above eosin staining was conducted to visualize the brain vessels. To avoid the risk of protein molecules being degraded after decrosslinking in the process of immunostainning, we did not conduct the immunostaining of Aβ, but, rather, conducted eosin staining which permits the fixation to be maintained during the LMD process. For the ADNC +/CAA + group, we used FFPE sections in which all vessels in the cortical regions were covered with Aβ in the immunohistochemical analysis using the neighboring sections.

LMD was performed using a Leica LMD6000 system (Leica Microsystems GmbH, Wetzlar, Germany). The Aβ deposition in CAA is exclusively in the cortex and not in the white matter. Eosin staining permits the cortex to be distinguished from white matter. To obtain the cortical parenchyma for use in a proteomics analysis, the cortical regions in the eosin-stained sections were randomly dissected until the total dissected area reached 15 mm^2^ (× 20 μm thickness = 0.3 mm^3^) (Fig. 1). To obtain cortical vessels for proteomics analysis, the vessels (except for large vessels) of the cortical regions in eosin-stained sections were randomly dissected until the total dissected area reached 15 mm^2^ (× 20 μm thickness = 0.3 mm^3^) (Fig. 1).

The dissected samples from the FFPE sections were directly collected in the cap of a tube (0.5 ml PCR Tube with Flat Cap, Thin-Wall, Non-Sterile, MaxyClear) containing 20 µl of phase-transfer surfactant (PTS) buffer in the LMD system. The collected 20 µl suspension of FFPE tissue was transferred to a PCT Micro Tube, 20 µl of PTS buffer was added to the cap and this solution was transferred to the PCT Micro Tube. This process was repeated twice to transfer a total of 60 µl of FFPE tissue suspension into the PCT Micro Tube, which was then covered with a PCT Micro Cap (50 µl size).

### Protein extraction from FFPE with a PCT treatment to prepare protein samples

All samples were incubated at 95 °C for 60 min in a block incubator (Eppendorf, Hamburg, Germany; with mixing at 1000 rpm). Thereafter, the FFPE samples were incubated in a Barocycler (NEP 2320 Enhanced; Pressure BioSciences, South Easton, MA) in two steps: first, 60 cycles of 95 s at 45,000 psi and 5 s at atmospheric pressure at 95 °C, and second, 50 cycles of 20 s at 45,000 psi and 15 s at atmospheric pressure at 95 °C.

### Protein digestion of FFPE samples with PCT treatment

Protein digestion was carried out as soon as possible after protein extraction. After centrifuging the above samples at 15,000 rpm and room temperature for 3 min, 40 μl aliquots of the supernatants were transferred to new PCT Micro Tubes with PCT Micro Caps (150 μl size) (Pressure BioSciences, South Easton, MA). Samples were reduced by treatment with 10 mM (±)-dithiothreitol (DTT) (Wako Pure Chemical Industries, Osaka, Japan) for 30 min at 25 °C, followed by alkylation with 40 mM iodoacetamide (IAA) (Wako Pure Chemical Industries, Osaka, Japan) in the dark at 25 °C. Samples were diluted by adjusting the buffer volume to 139 μl with 50 mM ammonium bicarbonate before the addition of the Protease Max surfactant (Promega, Madison, WI) and Lys-C (Wako Pure Chemical Industries, Osaka, Japan) at 0.04% final concentration and an enzyme/substrate ratio of 1:20, respectively. PCT-assisted Lys-C digestion was performed in the Barocycler at 37 °C using 60 cycles of 50 s at 45,000 psi, and 10 s at atmospheric pressure. Trypsin (Promega, Madison, WI) was then added at an enzyme/substrate ratio of 1:20 with a Protease Max surfactant. PCT-assisted trypsin digestion was performed in the Barocycler at 37 °C using 90 cycles of 50 s at 45,000 psi, and 10 s at atmospheric pressure. After enzyme digestion, SDC and SLS (PTS) were removed by liquid–liquid extraction using ethyl acetate. The aqueous phase was dried under vacuum and stored at − 80 °C until being desalted. Peptide samples dissolved in 0.1% trifluoroacetic acid/water were desalted with self-packed SDB-XD tips (3 M, Maplewood, MN), and the eluted peptide solution was again dried under a vacuum. Peptides samples were dissolved in 0.1% formic acid/2% acetonitrile/98% water, and their concentration was adjusted to 0.5 μg/μl based on the BCA assay (for parenchymal sample, but was not adjusted for vessel sample). The recovery from a 0.3 mm^3^ FFPE section was 8.22 ± 3.29 µg of peptide (mean ± SD).

### SWATH measurement by means of a nanoLC-TripleTOF5600 system

The SWATH-MS measurements and analyses were performed as previously described [3]. 1 µg peptide were applied to nano-LC ultra (Eksigent Technologies, Dublin, CA, USA) coupled with an electrospray-ionization Triple TOF 5600 mass spectrometer (SCIEX, Framingham, MA, USA).

### Data analysis for SWATH-based quantitative proteomics

Spectral alignment and data extraction from SWATH chromatograms (uploaded to the Peptide Atlas website with Identifier PASS01719) were performed with the SWATH Processing Micro App in Peakview (SCIEX) using an in-house spectral library (uploaded to the Peptide Atlas website with Identifier PASS01719) as previously described [3, 7]. According to the previously described procedure [7], unreliable peaks and peptides were removed based on the criteria of data selection and amino acid sequence-based peptide selection, and the peak areas at the peptide level were calculated as an average of those in the transition level after normalizing the differences in signal intensity between different transitions. The details of this procedure have been reported previously [7]. The peak areas of individual proteins were calculated as an average of those at the peptide level. For each protein, the peak areas for proteins in individual donors were finally divided by the average of protein peak areas in the capillary samples of five ADNC −/CAA − donors to obtain the relative protein expression level (Additional file 2: Table S1).

### Statistical analyses

All statistical analyses were performed under the null hypothesis, assuming that the means of the compared groups were equal. Comparison between two groups was performed by an unpaired two-tailed t-test, and the p value was adjusted by Benjamini-Hochberg (BH) correction in the case of multiple comparisons. For the correlation analysis, Spearman’s rank correlation coefficient and p value were calculated using Excel statistical software version 2010. If the p-value was less than 0.05, the difference was considered to be statistically significant and the null hypothesis was rejected. No formal power calculation was performed to estimate the required sample size. No randomization or blinding was performed in this study.

## Results

### Validation of the purity of the isolated capillaries

To confirm the purity of the cortical vessels isolated by LMD, we compared the expression levels of the glucose transporter 1 (GLUT1) which is an endothelial cell marker protein, and the Na^+^/H^+^ exchange regulatory cofactor NHE-RF2 (SLC9A3R2) which has been reported to be specifically expressed in cerebral blood vessels in the human protein atlas (https://www.proteinatlas.org/), among the capillary and parenchyma collected from the FFPE sections of the ADNC +/CAA +, ADNC +/CAA −, and ADNC −/CAA − groups. There were no significant differences in the protein expression levels in the collected capillaries among the three donor groups for all three proteins (p > 0.01) (Fig. 1). The expression levels of two proteins in the collected capillaries were significantly greater than those in the collected parenchyma for all three groups (p < 0.01) (Fig. 1). These results suggest that the cortical endothelial cells were enriched and the purity of the endothelial cells in the collected capillary samples was the same among ADNC +/CAA +, ADNC +/CAA −, and ADNC −/CAA − groups.

### Intergroup difference in protein expression levels among ADNC +/CAA +/H, ADNC +/CAA +/L, ADNC +/CAA −, and ADNC −/CAA − groups in the collected capillaries

The SWATH analysis of the collected capillary and parenchyma showed that 1255 proteins were quantified (Additional file 2: Table S1). Because of large individual differences in protein expression levels, for the ADNC +/CAA + group, the three donors with highly abundant Aβ in capillaries (donors 1, 2 and 3) were classified as “ADNC +/CAA +/H”, and the other three donors (donors 4, 5 and 6) were classified as “ADNC +/CAA +/L”. To identify the proteins specifically associated for CAA pathology, the proteins satisfying both the following (1) and (2) criteria were extracted; proteins that showed significant differences (BH-adjusted p < 0.05) in protein expression levels (1) between ADNC +/CAA +/H and ADNC +/CAA − groups, and (2) between ADNC +/CAA +/H and ADNC −/CAA − groups, for the collected capillaries. 17 proteins were extracted, and included 1 fibrosis related protein, 2 ROS related proteins, 2 antioxidant related proteins, 2 Akt related proteins, 4 caspase related proteins, and 6 other proteins (Table 2). Because of large individual differences in protein expression levels, further CAA-associated proteins were screened by the following correlation analysis.

**Table 2:** 17 proteins for which there were significant differences in protein expression levels in capillary samples between the Aβ abundant CAA (ADNC +/CAA +/H) and non-CAA groups (ADNC +/CAA − and ADNC −/CAA −)

Molecule name	Relative protein expression level normalized by the average in ADNC-/CAA- capillary samples				Benjamini & Hochberg (BH) adjusted p-value for the difference in protein expression levels between two groups in capillary sample					
	ADNC +/CAA +/H	ADNC +/CAA +/L	ADNC +/CAA-	ADNC-/CAA-	ADNC +/CAA +/H vs ADNC +/CAA +/L	ADNC +/CAA +/H vs ADNC +/CAA-	ADNC +/CAA +/H vs ADNC-/CAA-	ADNC +/CAA +/L vs ADNC +/CAA-	ADNC +/CAA +/L vs ADNC-/CAA-	ADNC +/CAA- vs ADNC-/CAA-
	MEAN ± SD	MEAN ± SD	MEAN ± SD	MEAN ± SD						
Fibrosis related protein										
DUSP3	0.373 ± 0.120	0.842 ± 0.344	0.800 ± 0.150	1.00 ± 0.25	0.180	0.0134*	0.0179*	0.851	0.618	0.243
ROS related protein										
PGM1	1.70 ± 0.05	1.09 ± 0.49	1.06 ± 0.42	1.00 ± 0.31	0.197	0.0459*	0.0163*	0.925	1.00	0.971
GSTO1	2.32 ± 0.45	1.50 ± 0.47	0.877 ± 0.461	1.00 ± 0.62	0.189	0.0289*	0.0394*	0.170	0.286	0.731
Antioxidant related protein										
GFAP	1.99 ± 0.21	1.85 ± 0.21	1.26 ± 0.39	1.00 ± 0.26	0.460	0.0289*	0.00585*	0.0512	0.00668*	0.287
PRDX6	1.98 ± 0.11	1.23 ± 0.39	0.998 ± 0.178	1.00 ± 0.41	0.0637	0.000393*	0.00712*	0.542	0.546	0.990
Akt related protein										
SNCG	0.215 ± 0.051	0.698 ± 0.457	1.05 ± 0.29	1.00 ± 0.37	0.286	0.00464*	0.0101*	0.407	0.444	0.798
PFDN2	0.467 ± 0.075	0.252 ± 0.031	1.18 ± 0.26	1.00 ± 0.43	0.0153*	0.00346*	0.0437*	0.00123*	0.0171*	0.454
Caspase related protein										
TLN2	0.430 ± 0.165	0.947 ± 0.306	0.959 ± 0.167	1.00 ± 0.15	0.124	0.0142*	0.0161*	0.952	0.944	1.00
ATP2A2	0.504 ± 0.077	0.780 ± 0.071	0.767 ± 0.125	1.00 ± 0.32	0.0615	0.0307*	0.0307*	0.865	0.221	0.246
CCT7	0.710 ± 0.063	0.902 ± 0.171	0.982 ± 0.130	1.00 ± 0.16	0.282	0.0441*	0.0357*	0.613	0.682	0.852
ELAVL1	0.548 ± 0.076	0.647 ± 0.150	1.04 ± 0.19	1.00 ± 0.31	0.436	0.0134*	0.0433*	0.0548	0.113	0.825
Others										
VARS	0.356 ± 0.088	0.640 ± 0.038	0.822 ± 0.233	1.00 ± 0.25	0.0204*	0.0138*	0.0110*	0.164	0.0281*	0.278
HGSNAT	1.89 ± 0.28	0.959 ± 0.515	0.804 ± 0.198	1.00 ± 0.27	0.104	0.00670*	0.0140*	0.762	0.905	0.342
PLPBP	0.810 ± 0.072	1.18 ± 0.06	1.20 ± 0.13	1.00 ± 0.09	0.00731*	0.0110*	0.0274*	0.862	0.0296*	0.0318*
PPA1	1.55 ± 0.20	1.04 ± 0.10	0.509 ± 0.332	1.00 ± 0.26	0.0269*	0.00918*	0.0325*	0.0458*	0.755	0.0380
SV2B	0.425 ± 0.045	0.829 ± 0.079	0.942 ± 0.121	1.00 ± 0.23	0.00309*	0.000818*	0.00455*	0.245	0.209	0.623
C2CD2L	0.497 ± 0.215	0.852 ± 0.434	1.08 ± 0.07	1.00 ± 0.15	0.409	0.0221*	0.0347*	0.472	0.592	0.536

The 17 proteins listed in this table satisfy both the following (1) and (2) criteria; proteins that showed significant differences in protein expression levels (1) between ADNC +/CAA +/H and ADNC +/CAA − groups, and (2) between ADNC +/CAA +/H and ADNC −/CAA − groups, for the collected capillaries

ADNC +/CAA +/H donors were three ADNC +/CAA + donors with highly abundant Aβ in capillaries, and ADNC +/CAA +/L donors were three ADNC +/CAA + donors with less abundant Aβ in capillaries

*ADNC* Alzheimer’s disease neuropathologic change; *CAA* cerebral amyloid angiopathy

^*^BH-adjusted p < 0.05, significantly different between two groups

### Correlation of protein expression levels with Aβ or Collagen alpha-2(VI) chain (COL6A2)

Assuming that the amount of Aβ present in the blood vessels is indicative of the severity of the disease, there was a 120-fold difference in severity among the six patients in ADNC +/CAA + groups, suggesting a high degree of individual variation (Table 1). We hypothesized that the proteins associated with disease would change in correlation with the severity of the disease. COL6A2, one of the proteins that was quantified in this study, has been reported to accumulate in blood vessels in ADNC +/CAA + patients, as does Aβ [8]. In this study, Aβ and COL6A2 were used as indicators of the severity of the disease, and the correlation between these molecules and each other protein was used to narrow down the candidate proteins that could be potentially associated with CAA pathology. A Spearman's rank correlation analysis for only six ADNC +/CAA + samples did not allow us to identify promising proteins because this analysis failed to successfully exclude the proteins whose expression levels did not differ from those in the ADNC −/CAA − group. Therefore, we extracted candidate proteins using the two selections as follows; (1) proteins that showed more than a 1.5-fold difference in average expression levels between six ADNC +/CAA + and five ADNC −/CAA − donors for the collected capillaries, and (2) proteins that are significantly (p < 0.05) correlated with Aβ or COL6A2 in 11 donors (6 ADNC +/CAA + and 5 ADNC −/CAA − donors) based on Spearman’s rank correlation analysis. There were 43 proteins that met these two criteria and were significantly correlated with Aβ (Table 3) and 46 proteins that were significantly correlated with COL6A2 (Additional file 2: Table S2). To determine whether the changes in the expression of these proteins occur only in the capillaries of ADNC +/CAA + patients (and not in the capillaries of ADNC +/CAA − patients or in the parenchyma of both ADNC +/CAA + and ADNC +/CAA − patients), we conducted the Spearman’s correlation analysis for these proteins using the data obtained for the capillaries of ADNC +/CAA − patients and the parenchyma. In the capillaries, only 5 out of 43 proteins were significantly correlated with Aβ (Table 3) and only 8 out of 46 proteins with COL6A2 (Additional file 2: Table S2) in the Spearman’s correlation analysis using the 10 donors (5 ADNC +/CAA − and 5 ADNC −/CAA − donors). In the parenchyma, only 1 out of 43 proteins was significantly correlated with Aβ (Table 3) and only 2 out of 46 proteins were significantly correlated with COL6A2 (Additional file 2: Table S2) in the Spearman's correlation analysis using the 11 donors (6 ADNC +/CAA + and 5 ADNC −/CAA − donors). In the Spearman’s correlation analysis using parenchyma from 10 doners (5 ADNC +/CAA − and 5 ADNC −/CAA − donors), there were no proteins (out of 43 proteins) that were significantly correlated with Aβ (Table 3), and only 8 out of 46 proteins were significantly correlated with COL6A2 (Additional file 2: Table S2).

**Table 3:** Spearman’s rank correlation with Aβ to extract CAA-specific and vascular-specific pathological changes

	Capillary				Parenchyma			
	11 donors including CAA (ADNC +/CAA + & ADNC −/CAA −)		10 donors including AD (ADNC +/CAA- & ADNC −/CAA −)		11 donors including CAA (ADNC +/CAA + & ADNC − −/CAA −)		10 donors including AD (ADNC +/CAA − & ADNC −/CAA −)	
	ρ	p	ρ	p	ρ	p	ρ	p
Aβ	1.00	–	1.00	–	1.00	–	1.00	–
**ECM related protein**								
COL6A1	0.855	8.07E−04**	0.467	1.74E−01	0.273	4.17E−01	0.309	3.85E−01
FBN1	0.800	3.11E−03**	0.515	1.28E−01	− 0.0636	8.53E−01	0.527	1.17E−01
COL6A2	0.782	4.47E−03**	0.370	2.93E−01	− 0.0909	7.90E−01	0.406	2.44E−01
COL6A3	0.691	1.86E−02*	0.297	4.05E−01	− 0.100	7.70E−01	0.261	4.67E−01
**Cell adhesion related protein**								
CD44	0.645	3.20E−02*	0.661	3.76E−02*	0.327	3.26E−01	0.539	1.08E−01
HEPACAM	0.618	4.26E−02*	0.418	2.29E−01	− 0.536	8.90E−02	0.333	3.47E−01
PCBP1	− 0.809	2.56E−03**	− 0.624	5.37E−02	− 0.264	4.33E−01	0.612	6.00E−02
**Fibrosis related protein**								
HSP90A	0.764	6.23E−03**	− 0.0667	8.55E−01	0.227	5.02E−01	0.236	5.11E−01
LDHA	0.727	1.12E−02*	0.164	6.51E−01	0.00909	9.79E−01	0.188	6.03E−01
S100A6	0.664	2.60E−02*	0.115	7.51E−01	0.264	4.33E−01	− 0.273	4.46E−01
PML	0.636	3.53E−02*	0.527	1.17E−01	− 0.0818	8.11E−01	0.273	4.46E−01
DUSP3	− 0.782	4.47E−03**	− 0.139	7.01E−01	− 0.445	1.70E−01	0.103	7.77E−01
**ROS related protein**								
APOE	0.827	1.68E−03**	0.321	3.65E−01	0.582	6.04E−02	0.467	1.74E−01
GNG12	0.700	1.65E−02*	0.673	3.30E−02*	− 0.00909	9.79E−01	0.382	2.76E−01
GSTO1	0.673	2.33E−02*	− 0.0545	8.81E−01	0.191	5.74E−01	− 0.273	4.46E−01
IGHG1	0.655	2.89E−02*	0.309	3.85E−01	0.0818	8.11E−01	0.370	2.93E−01
**Antioxidant related protein**								
GFAP	0.864	6.12E−04**	0.200	5.80E−01	0.509	1.10E−01	− 0.224	5.33E−01
PRDX2	0.727	1.12E−02*	0.139	7.01E−01	− 0.182	5.93E−01	− 0.224	5.33E−01
CYB5R3	0.655	2.89E−02*	0.527	1.17E−01	− 0.136	6.89E−01	0.164	6.51E−01
CLU	0.655	2.89E−02*	0.176	6.27E−01	0.509	1.10E−01	− 0.0303	9.34E−01
PRDX6	0.627	3.88E−02*	− 0.236	5.11E−01	0.218	5.19E−01	− 0.0182	9.60E−01
**Akt related protein**								
PDHX	− 0.791	3.75E−03**	− 0.127	7.26E−01	− 0.0818	8.11E−01	0.309	3.85E−01
NCSTN	− 0.782	4.47E−03**	− 0.285	4.25E−01	− 0.0182	9.58E−01	− 0.00606	9.87E−01
TPD52L2	− 0.691	1.86E−02*	− 0.333	3.47E−01	− 0.0818	8.11E−01	− 0.0909	8.03E−01
PSMC2	− 0.664	2.60E−02*	− 0.370	2.93E−01	0.0182	9.58E−01	− 0.297	4.05E−01
**Caspase related protein**								
HEBP1	0.691	1.86E−02*	0.697	2.51E−02*	− 0.455	1.60E−01	0.0788	8.29E−01
CA1	0.645	3.20E−02*	0.139	7.01E−01	− 0.0455	8.94E−01	0.345	3.28E−01
TUBB2B	− 0.845	1.05E−03**	− 0.345	3.28E−01	0.109	7.50E−01	0.418	2.29E−01
ACOX1	− 0.882	3.30E−04**	− 0.770	9.22E−03**	− 0.327	3.26E−01	− 0.285	4.25E−01
ATP2A2	− 0.873	4.55E−04**	− 0.382	2.76E−01	− 0.409	2.12E−01	0.333	3.47E−01
PTGES3	− 0.736	9.76E−03**	− 0.00606	9.87E−01	− 0.364	2.72E−01	0.455	1.87E−01
PRKAR2B	− 0.736	9.76E−03**	− 0.455	1.87E−01	0	–	0.273	4.46E−01
TUBB4A	− 0.718	1.28E−02*	− 0.200	5.80E−01	− 0.309	3.55E−01	0.442	2.00E−01
PPP2R2A	− 0.691	1.86E−02*	− 0.00606	9.87E−01	− 0.245	4.67E−01	0.358	3.10E−01
TUBB6	− 0.664	2.60E−02*	− 0.382	2.76E−01	0.145	6.70E−01	0.152	6.76E−01
**Others**								
KANK2	0.773	5.30E−03**	0.0909	8.03E−01	0.618	4.26E−02*	− 0.0788	8.29E−01
VARS	− 0.945	1.12E−05**	− 0.661	3.76E−02*	− 0.364	2.72E−01	− 0.0303	9.34E−01
ACTN2	− 0.818	2.08E−03**	− 0.479	1.62E−01	− 0.218	5.19E−01	0.0788	8.29E−01
EXOC6B	− 0.800	3.11E−03**	− 0.188	6.03E−01	− 0.327	3.26E−01	− 0.176	6.27E−01
HSPH1	− 0.709	1.46E−02*	− 0.261	4.67E−01	− 0.227	5.02E−01	− 0.0303	9.34E−01
SV2B	− 0.709	1.46E−02*	− 0.382	2.76E−01	− 0.445	1.70E−01	0.248	4.89E−01
ETFA	− 0.700	1.65E−02*	0.224	5.33E−01	0.0182	9.58E−01	0.0182	9.60E−01
FARSA	− 0.691	1.86E−02*	− 0.309	3.85E−01	0	–	− 0.0788	8.29E−01

The 43 proteins listed in this table (except for Aβ) were chosen by the two selections as follows; (1) proteins that showed more than 1.5-fold difference in the average expression levels between six ADNC +/CAA + and five ADNC −/CAA − donors for the collected capillaries, and (2) proteins that are significantly (p < 0.05) correlated with Aβ in the capillary samples of 11 donors (6 ADNC +/CAA + and 5 ADNC −/CAA − donors) based on Spearman’s rank correlation analysis. The ρ and p values of Spearman’s rank correlation were listed for four conditions; 11 donors’ capillaries (6 ADNC +/CAA + and 5 ADNC −/CAA − donors), 10 donors’ capillaries (5 ADNC +/CAA − and 5 ADNC −/CAA − donors), 11 donors’ parenchyma (6 ADNC +/CAA + and 5 ADNC −/CAA − donors), and 10 donors’ parenchyma (5 ADNC +/CAA − and 5 ADNC −/CAA − donors)

*p < 0.05

**p < 0.01, significantly correlation with Aβ

### Extracellular matrix (ECM) component proteins

**Fig. 2:**
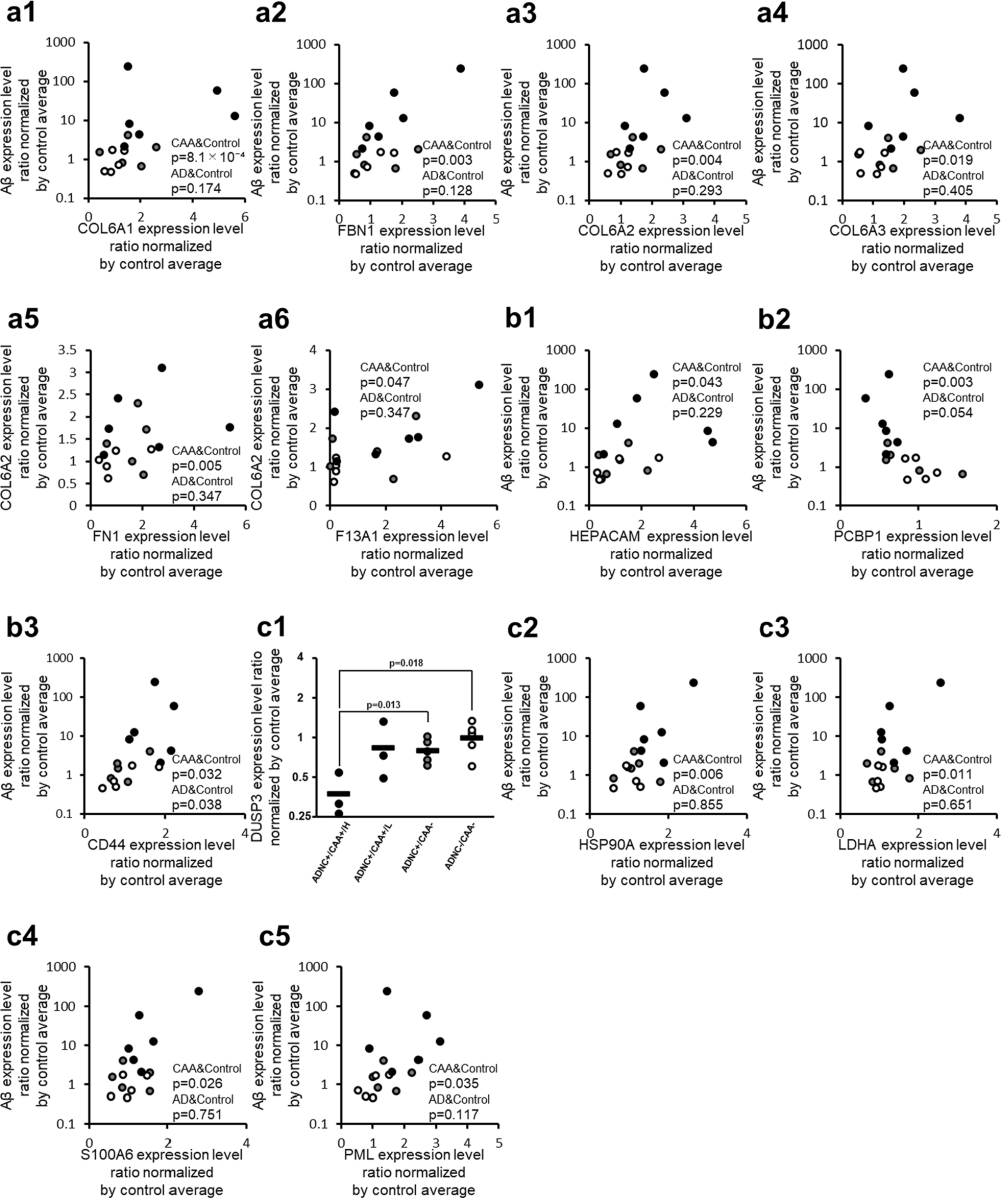
CAA-specific changes in protein expression levels of fibrosis-associated molecules in the collected capillaries. For fibrosis-associated molecules listed in Table 2, the graph showing their intergroup comparisons is preferentially presented in this figure. For fibrosis-associated molecules not listed in Table 2 but listed in Table 3 or Additional file 2: Table S2, the correlation graphs with Aβ are shown in this figure if the protein showed a significant correlation both with Aβ (Table 3) and COL6A2 (Additional file 2: Table S2). **a** ECM-related proteins, **b** Cell-ECM adhesion-related proteins, **c** TGF-β signal-related proteins. The data for protein expression levels for individual donors (normalized by average of protein expression levels in ADNC −/CAA − capillaries as described in the "Materials and methods" section) were taken from Additional file 2: Table S1. Black plot, ADNC +/CAA + (CAA); Gray plot, ADNC +/CAA − (AD); White plot, ADNC −/CAA − (Control). For the ADNC +/CAA + group, the three donors with highly abundant Aβ in capillaries (donors 1, 2 and 3) were classified as “ADNC +/CAA +/H”, and the other three donors (donors 4, 5 and 6) were classified as “ADNC +/CAA +/L”. The p-values were taken from Tables 2, 3 or Additional file 2: Table S2. For the correlation graphs, the p-values of “CAA&Control” and “AD&Control” represent the ones in Spearman’s rank correlation using 11 donors (ADNC +/CAA + and ADNC −/CAA −) and 10 donors (ADNC +/CAA − and ADNC −/CAA −), respectively

Among the proteins constituting the ECM and contributing to its stabilization, the proteins that showed a significant (p < 0.05) positive correlation with Aβ according to Spearman’s rank correlation coefficient using 11 ADNC +/CAA + and ADNC −/CAA − donors were Collagen alpha-1(VI) chain (COL6A1; p = 8.07 × 10^–4^), Fibrillin-1 (FBN1; p = 3.11 × 10^–3^), COL6A2 (p = 4.47 × 10^–3^), and Collagen alpha-3(VI) chain (COL6A3; p = 1.86 × 10^–2^) in capillary samples (Fig. 2 and Table 3). No protein showed a negative correlation. Similarly, the proteins that showed a significant (p < 0.05) positive correlation with COL6A2 in 11 ADNC +/CAA + and ADNC −/CAA − donors were COL6A3 (p = 8.40 × 10^–8^), COL6A1 (p = 3.30 × 10^–4^), Fibronectin (FN1; p = 5.30 × 10^–3^), FBN1 (p = 9.76 × 10^–3^), Coagulation factor XIII A chain (F13A1; p = 4.67 × 10^–2^) and no protein in the capillary samples showed a negative correlation (Fig. 2 and Additional file 2: Table S2). None of these proteins showed a significant correlation with Aβ in the Spearman’s rank correlation analysis using 10 ADNC +/CAA − and ADNC −/CAA − donors in capillary samples (Fig. 2). For the 6 ECM-related proteins shown in Fig. 2, the graphs of intergroup comparisons for the collected capillaries were also shown in Additional file 1: Figure S2, but no significant difference was observed between ADNC +/CAA +/H and ADNC +/CAA − groups (BH-adjusted p > 0.05).

### Adhesion molecules

Among the proteins involved in adhesion between the cell and ECM, the proteins that showed a significant (p < 0.05) positive correlation with Aβ according to Spearman’s rank correlation coefficient using 11 ADNC +/CAA + and ADNC −/CAA − donors were CD44 antigen (CD44; p = 3.20 × 10^−2^) and Hepatocyte cell adhesion molecule (HEPACAM; p = 4.26 × 10^−2^) in capillary samples (Fig. 2 and Table 3). A negative correlation was observed in the case of the Poly(rC)-binding protein 1 (PCBP1; p = 2.56 × 10^−3^). For these three molecules, the p-values for the correlations were lower than those using 10 ADNC +/CAA − and ADNC −/CAA − donors (Fig. 2). Similarly, the protein that showed a significant (p < 0.05) positive correlation with COL6A2 in 11 ADNC +/CAA + and ADNC −/CAA − donors was CD44 (p = 1.86 × 10^−2^). PCBP1 (p = 8.43 × 10^−3^) showed a negative correlation in the capillary samples (Additional file 2: Table S2). For the 3 adhesion molecules shown in Fig. 2, the graphs of intergroup comparisons for the collected capillaries were also shown in Additional file 1: Figure S2, but no significant difference was observed between ADNC +/CAA +/H and ADNC +/CAA − groups (BH-adjusted p > 0.05).

### Fibrosis-associated proteins

The protein expression levels of Dual specificity protein phosphatase 3 (DUSP3) in ADNC +/CAA +/H donors were significantly smaller than those in ADNC +/CAA − and ADNC −/CAA − donors (BH-adjusted p < 0.05) (Fig. 2 and Table 2). Among the proteins involved in fibrosis, the proteins that showed a significant (p < 0.05) positive correlation with Aβ according to Spearman's rank correlation coefficient using 11 ADNC +/CAA + and ADNC −/CAA − donors were Heat shock protein HSP 90-alpha (HSP90A; p = 6.23 × 10^−3^), L-lactate dehydrogenase A chain (LDHA; p = 1.12 × 10^−2^), Protein S100-A6 (S100A6; p = 2.60 × 10^−2^), and Protein PML (PML; p = 3.53 × 10^−2^) in capillary samples (Fig. 2 and Table 3). A negative correlation was observed for DUSP3 (p = 4.47 × 10^−3^). None of these proteins showed a significant correlation with Aβ in the Spearman’s rank correlation analysis using 10 ADNC +/CAA − and ADNC −/CAA − donors in capillary samples (Fig. 2). Similarly, the proteins that showed a significant (p < 0.05) positive correlation with COL6A2 in 11 ADNC +/CAA + and ADNC −/CAA − donors were S100A6 (p = 4.55 × 10^−4^), PML (p = 8.45 × 10^−3^), LDHA (p = 3.53 × 10^−2^), and HSP90A (p = 3.53 × 10^−2^). DUSP3 (p = 3.20 × 10^−2^) showed a negative correlation in the capillary samples (Additional file 2: Table S2). For the 4 TGF-β signal-related proteins shown with correlation graphs in Fig. 2, the graphs of intergroup comparisons for the collected capillaries were also shown in Additional file 1: Figure S2, but no significant difference was observed between ADNC +/CAA +/H and ADNC +/CAA- groups (BH-adjusted p > 0.05).

### Oxidative stress-related proteins

**Fig. 3:**
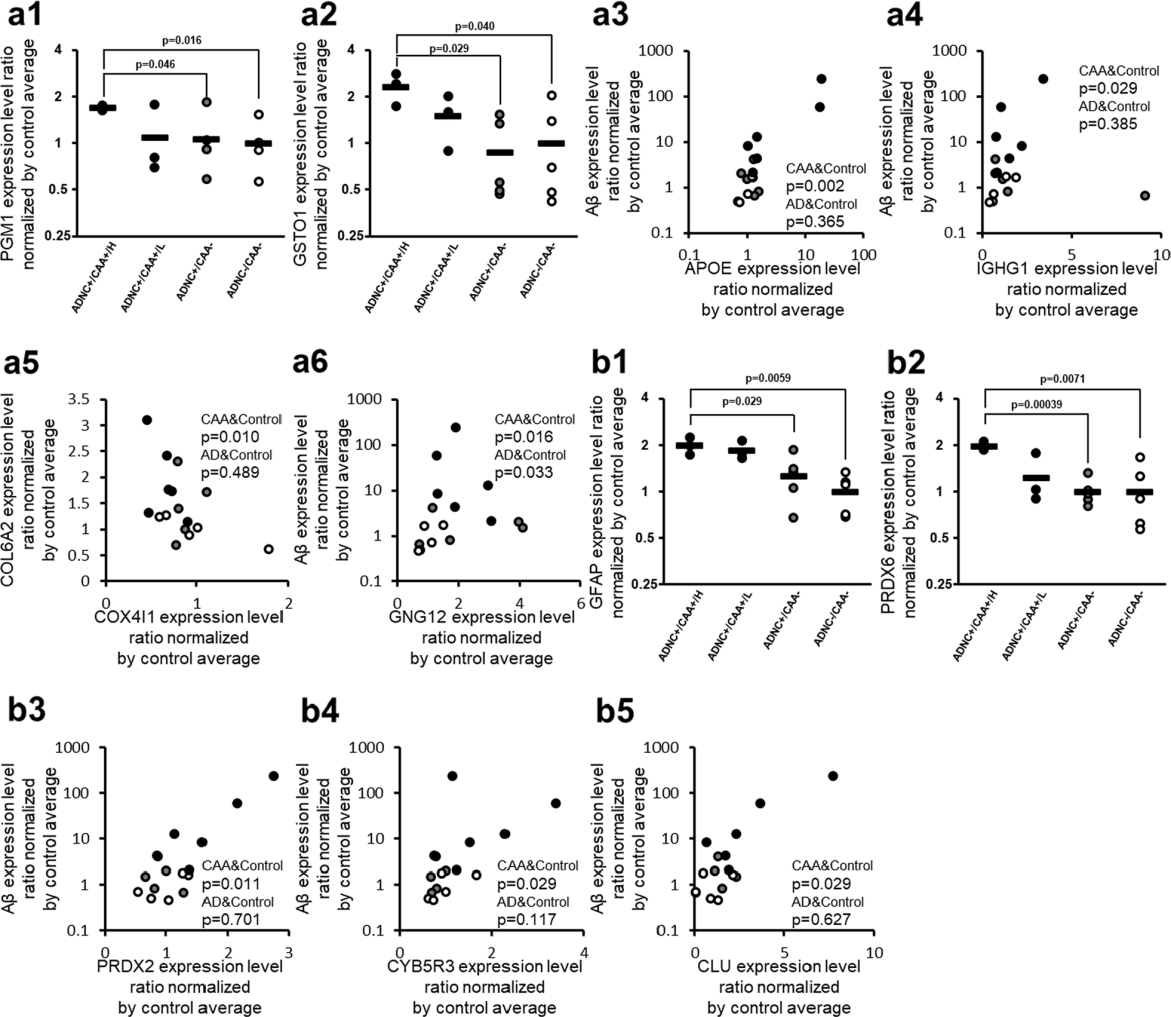
CAA-specific changes in protein expression levels of oxidative stress-associated molecules in the collected capillaries. For oxidative stress-associated proteins listed in Table 2, the graphs showing their intergroup comparisons are preferentially presented in this figure. For oxidative stress-associated proteins not listed in Table 2 but listed in Table 3 or Additional file 2: Table S2, the correlation graphs with Aβ are shown in this figure if the protein showed a significant correlation both with Aβ (Table 3) and COL6A2 (Additional file 2: Table S2). **a** ROS-related proteins, and **b** Antioxidant-related proteins. The data for protein expression levels for individual donors (normalized by average of protein expression levels in ADNC −/CAA − capillaries as described in the "Materials and methods" section) were taken from Additional file 2: Table S1. Black plot, ADNC +/CAA + (CAA); Gray plot, ADNC +/CAA − (AD); White plot, ADNC −/CAA − (Control). For the ADNC +/CAA + group, the three donors with highly abundant Aβ in capillaries (donors 1, 2 and 3) were classified as “ADNC +/CAA +/H”, and the other three donors (donors 4, 5 and 6) were classified as “ADNC +/CAA +/L”. The p-values were taken from Tables 2, 3 or Additional file 2: Table S2. For the correlation graphs, the p-values of “CAA&Control” and “AD&Control” represent the ones in Spearman’s rank correlation using 11 donors (ADNC +/CAA + and ADNC −/CAA −) and 10 donors (ADNC +/CAA − and ADNC −/CAA −), respectively

The protein expression levels of Phosphoglucomutase-1 (PGM1) and Glutathione S-transferase omega-1 (GSTO1) in ADNC +/CAA +/H donors were significantly greater than those in ADNC +/CAA − and ADNC −/CAA − donors (BH-adjusted p < 0.05) (Fig. 3 and Table 2). Among the proteins involved in oxidative stress, the proteins that showed a significant (p < 0.05) positive correlation with Aβ according to Spearman’s rank correlation coefficient using 11 ADNC +/CAA + and ADNC −/CAA − donors were Apolipoprotein E (APOE; p = 1.68 × 10^−3^), Guanine nucleotide-binding protein G(I)/G(S)/G(O) subunit gamma-12 (GNG12; p = 1.65 × 10^−2^), GSTO1 (p = 2.33 × 10^−2^), and Immunoglobulin heavy constant gamma 1 (IGHG1; p = 2.89 × 10^−2^) in capillary samples (Fig. 3 and Table 3). Only GNG12 (p = 3.30 × 10^−2^) showed a significant positive correlation with Aβ in the Spearman’s rank correlation analysis using 10 ADNC +/CAA − and ADNC −/CAA − donors in capillary samples (Fig. 3), but the p-value for GNG12 was greater than those obtained when 11 ADNC +/CAA + and ADNC −/CAA − donors were examined. Similarly, the proteins that showed a significant (p < 0.05) positive correlation with COL6A2 in 11 ADNC +/CAA + and ADNC −/CAA − donors were APOE (p = 3.30 × 10^−4^), GSTO1 (p = 4.47 × 10^−3^), and GNG12 (p = 4.67 × 10^−2^). Mitochondrial cytochrome c oxidase subunit 4 isoform 1 (COX4I1; p = 9.76 × 10^−3^) showed a negative correlation in capillary samples (Fig. 3 and Additional file 2: Table S2). None of these proteins showed a significant correlation with COL6A2 in the Spearman’s rank correlation analysis using 10 ADNC +/CAA − and ADNC −/CAA − donors in capillary samples (Additional file 2: Table S2). For the 4 ROS-related proteins shown with correlation graphs in Fig. 3, the graphs of intergroup comparisons for the collected capillaries were also shown in Additional file 1: Figure S3, but no significant difference was observed between ADNC +/CAA +/H and ADNC +/CAA − groups (BH-adjusted p > 0.05).

### Antioxidant proteins

The protein expression levels of Glial fibrillary acidic protein (GFAP) and Peroxiredoxin-6 (PRDX6) in ADNC +/CAA +/H donors were significantly greater than those in ADNC +/CAA − and ADNC −/CAA − donors (BH-adjusted p < 0.05) (Fig. 3 and Table 2). Among the antioxidant proteins, the proteins that showed a significant (p < 0.05) positive correlation with Aβ according to Spearman’s rank correlation coefficient using 11 ADNC +/CAA + and ADNC −/CAA − donors were GFAP (p = 6.12 × 10^−4^), Peroxiredoxin-2 (PRDX2; p = 1.12 × 10^−2^), NADH-cytochrome b5 reductase 3 (CYB5R3; p = 2.89 × 10^−2^), Clusterin (CLU; p = 2.89 × 10^−2^), and PRDX6 (p = 3.88 × 10^−2^) in capillary samples (Fig. 3 and Table 3). Similarly, the proteins that showed a significant (p < 0.05) positive correlation with COL6A2 in 11 ADNC +/CAA + and ADNC −/CAA − donors were CLU (p = 3.75 × 10^−3^), GFAP (p = 1.12 × 10^−2^), PRDX6 (p = 1.12 × 10^−2^), and CYB5R3 (p = 1.65 × 10^−2^) (Additional file 2: Table S2). None of these proteins showed a significant correlation with Aβ or COL6A2 in the Spearman’s rank correlation analysis using 10 ADNC +/CAA − and ADNC −/CAA − donors in capillary samples (Fig. 3, Table 3, and Additional file 2: Table S2). For the 3 antioxidant-related proteins shown with correlation graphs in Fig. 3, the graphs of intergroup comparisons for the collected capillaries were also shown in Additional file 1: Figure S3, but no significant difference was observed between ADNC +/CAA +/H and ADNC +/CAA − groups (BH-adjusted p > 0.05).

### AKT signal proteins

**Fig. 4:**
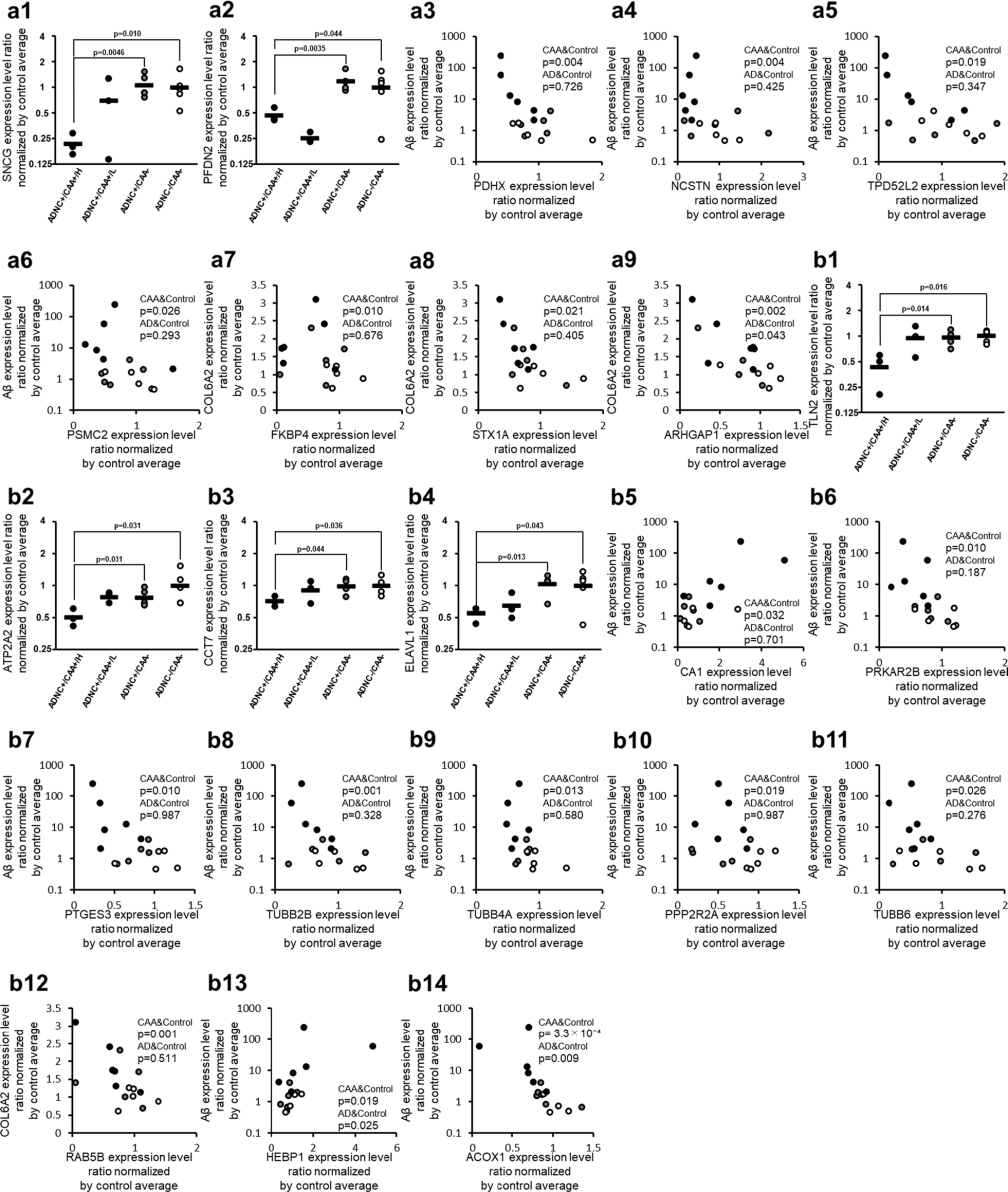
CAA-specific changes in protein expression levels of apoptosis-associated molecules in the collected capillaries. For apoptosis-associated proteins listed in Table 2, the graphs showing their intergroup comparisons are preferentially presented in this figure. For apoptosis-associated proteins not listed in Table 2 but listed in Table 3 or Additional file 2: Table S2, the correlation graphs with Aβ are shown in this figure if the protein showed a significant correlation both with Aβ (Table 3) and COL6A2 (Additional file 2: Table S2). **a** AKT signal-related proteins, and **b** Caspase signal-related proteins. The data for protein expression levels for individual donors (normalized by average of protein expression levels in ADNC −/CAA − capillaries as described in the "Materials and methods" section) were taken from Additional file 2: Table S1. Black plot, ADNC +/CAA + (CAA); Gray plot, ADNC +/CAA − (AD); White plot, ADNC −/CAA − (Control). For the ADNC +/CAA + group, the three donors with highly abundant Aβ in capillaries (donors 1, 2 and 3) were classified as “ADNC +/CAA +/H”, and the other three donors (donors 4, 5 and 6) were classified as “ADNC +/CAA +/L”. The p-values were taken from Tables 2, 3 or Additional file 2: Table S2. For the correlation graphs, the p-values of “CAA&Control” and “AD&Control” represent the ones in Spearman’s rank correlation using 11 donors (ADNC +/CAA + and ADNC −/CAA −) and 10 donors (ADNC +/CAA − and ADNC −/CAA −), respectively

The protein expression levels of Gamma-synuclein (SNCG) and Prefoldin subunit 2 (PFDN2) in ADNC +/CAA +/H donors were significantly smaller than those in ADNC +/CAA − and ADNC −/CAA − donors (BH-adjusted p < 0.05) (Fig. 4 and Table 2). Among the proteins involved in AKT signaling, there was no protein that showed a significant positive correlation with Aβ or COL6A2 in Spearman’s rank correlation analysis using 11 ADNC +/CAA + and ADNC −/CAA − donors in capillary samples (Table 3 and Additional file 2: Table S2). In contrast, the proteins that showed a significant negative correlation with Aβ were Mitochondrial pyruvate dehydrogenase protein X component (PDHX; p = 3.75 × 10^−3^), Nicastrin (NCSTN; p = 4.47 × 10^−3^), Tumor protein D54 (TPD52L2; p = 1.86 × 10^−2^), and 26S proteasome regulatory subunit 7 (PSMC2; p = 2.60 × 10^−2^) (Fig. 4 and Table 3). The proteins that showed a significant negative correlation with COL6A2 were NCSTN (p = 1.05 × 10^−3^), Rho GTPase-activating protein 1 (ARHGAP1; p = 2.08 × 10^−3^), Peptidyl-prolyl cis–trans isomerase FKBP4 (FKBP4; p = 9.76 × 10^−3^), Syntaxin-1A (STX1A; p = 2.08 × 10^−2^), and PDHX (p = 2.08 × 10^−2^) in capillary samples (Fig. 4 and Additional file 2: Table S2). Only ARHGAP1 (p = 4.25 × 10^−2^) showed a significant negative correlation with COL6A2 in the Spearman’s rank correlation analysis using 10 ADNC +/CAA − and ADNC −/CAA − donors in capillary samples (Fig. 4), but the p-value of ARHGAP1 was greater than those for 11 ADNC +/CAA + and ADNC −/CAA − donors. For the 7 AKT signal-related proteins shown with correlation graphs in Fig. 4, the graphs of intergroup comparisons for the collected capillaries were also shown in Additional file 1: Figure S4. PDHX and TPD52L2 showed significant decreases in protein expression levels in ADNC +/CAA +/H donors compared to ADNC +/CAA − donors, whereas no significant difference was observed between ADNC +/CAA +/H and ADNC +/CAA − groups for the other 5 proteins (BH-adjusted p > 0.05).

### Caspase signal proteins

The protein expression levels of Talin-2 (TLN2), Sarcoplasmic/endoplasmic reticulum calcium ATPase 2 (ATP2A2), T-complex protein 1 subunit eta (CCT7) and ELAV-like protein 1 (ELAVL1) in ADNC +/CAA +/H donors were significantly smaller than those in ADNC +/CAA − and ADNC −/CAA − donors (BH-adjusted p < 0.05) (Fig. 4 and Table 2). Among the proteins involved in caspase signaling, the proteins that showed a significant (p < 0.05) positive correlation with Aβ according to Spearman's rank correlation coefficient using 11 ADNC +/CAA + and ADNC −/CAA − donors were Heme-binding protein 1 (HEBP1; p = 1.86 × 10^−2^), and Carbonic anhydrase 1 (CA1; p = 3.20 × 10^−2^) in capillary samples (Fig. 4 and Table 3). In contrast, the proteins that showed a significant negative correlation with Aβ were Peroxisomal acyl-coenzyme A oxidase 1 (ACOX1; p = 3.30 × 10^−4^), ATP2A2 (p = 4.55 × 10^−4^), Tubulin beta-2B chain (TUBB2B; p = 1.05 × 10^−3^), Prostaglandin E synthase 3 (PTGES3; p = 9.76 × 10^−3^), cAMP-dependent protein kinase type II-beta regulatory subunit (PRKAR2B; p = 9.76 × 10^−3^), Tubulin beta-4A chain (TUBB4A; p = 1.28 × 10^−2^), Serine/threonine-protein phosphatase 2A 55 kDa regulatory subunit B alpha isoform (PPP2R2A; p = 1.86 × 10^−2^), and Tubulin beta-6 chain (TUBB6; p = 2.60 × 10^−2^) (Fig. 4 and Table 3). The proteins that showed a significant negative correlation with COL6A2 were TUBB4A (p = 2.21 × 10^−5^), Ras-related protein Rab-5B (RAB5B; p = 1.33 × 10^−3^), ACOX1 (p = 8.45 × 10^−3^), ATP2A2 (p = 1.12 × 10^−2^), PPP2R2A (p = 1.86 × 10^−2^), PTGES3 (p = 2.33 × 10^−2^), PRKAR2B (p = 3.53 × 10^−2^), and TUBB2B (p = 3.88 × 10^−2^) (Fig. 4 and Additional file 2: Table S2). Spearman's rank correlation analysis using 10 ADNC +/CAA − and ADNC −/CAA − donors in capillary samples showed significant correlations only for HEBP1 (with Aβ, p = 2.51 × 10^−2^), ACOX1 (with Aβ, p = 9.22 × 10^−3^) and TUBB2B (with COL6A2, p = 3.88 × 10^−2^) (Fig. 4 and Additional file 2: Table S2). For the 10 caspase signal-related proteins shown with correlation graphs in Fig. 4, the graphs of intergroup comparisons for the collected capillaries were also shown in Additional file 1: Figure S4, but no significant difference was observed between ADNC +/CAA +/H and ADNC +/CAA − groups (BH-adjusted p > 0.05).

### Other proteins

The protein expression levels of Valine–tRNA ligase (VARS), Heparan-alpha-glucosaminide N-acetyltransferase (HGSNAT), Pyridoxal phosphate homeostasis protein (PLPBP), Soluble inorganic pyrophosphatase 1 (PPA1), Synaptic vesicle glycoprotein 2B (SV2B), and Phospholipid transfer protein C2CD2L (C2CD2L) in ADNC +/CAA +/H donors were significantly different from those in ADNC +/CAA − and ADNC −/CAA − donors (BH-adjusted p < 0.05) (Additional file 1: Figure S1 and Table 2). Among the proteins not associated with the molecular mechanisms alluded to above, the protein that showed a significant (p < 0.05) positive correlation with Aβ according to Spearman's rank correlation coefficient using 11 ADNC +/CAA + and ADNC −/CAA − donors was KN motif and ankyrin repeat domain-containing protein 2 (KANK2; p = 5.30 × 10^−3^) in capillary samples (Table 3 and Additional file 1: Figure S1). In contrast, the proteins that showed a significant negative correlation with Aβ were VARS (p = 1.12 × 10^−5^), Alpha-actinin-2 (ACTN2; p = 2.08 × 10^−3^), Exocyst complex component 6B (EXOC6B; p = 3.11 × 10^−3^), Heat shock protein 105 kDa (HSPH1; p = 1.46 × 10^−2^), SV2B (p = 1.46 × 10^−2^), Mitochondrial electron transfer flavoprotein subunit alpha (ETFA; p = 1.65 × 10^−2^), and Phenylalanine–tRNA ligase alpha subunit (FARSA; p = 1.86 × 10^−2^) (Table 3 and Additional file 1: Figure S1). The proteins that showed a significant positive correlation with COL6A2 were KANK2 (p = 4.47 × 10^−3^), and Proteasome activator complex subunit 1 (PSME1; p = 3.53 × 10^−2^), while HSPH1 (p = 3.97 × 10^−5^), SV2B (p = 2.33 × 10^−4^), EXOC6B (p = 6.12 × 10^−4^), VARS (p = 6.12 × 10^−4^), ACTN2 (p = 8.45 × 10^−3^), Noelin (OLFM1; p = 9.76 × 10^−3^), NADH dehydrogenase 1 alpha subcomplex subunit 2 (NDUFA2; p = 1.65 × 10^−2^), NADH dehydrogenase 1 alpha subcomplex subunit 8 (NDUFA8; p = 2.33 × 10^−2^), FARSA (p = 2.89 × 10^−2^), Ras-related protein Rab-3A (RAB3A; p = 3.88 × 10^−2^), and Polyadenylate-binding protein 4 (PABPC4; p = 4.26 × 10^−2^) showed a significant negative correlation with COL6A2 according to Spearman's rank correlation coefficient using 11 ADNC +/CAA + and ADNC −/CAA − donors in capillary samples (Additional file 2: Table S2 and Additional file 1: Figure S1). For the 12 unclassified other proteins shown with correlation graphs in Additional file 1: Figure S1, the graphs of intergroup comparisons for the collected capillaries were also shown in Additional file 1: Figure S5, but no significant difference was observed between ADNC +/CAA +/H and ADNC +/CAA − groups (BH-adjusted p > 0.05).

### Discussion

After isolating the Aβ-accumulating cortical vessels using LMD from FFPE sections of ADNC +/CAA + patients, we combined the PCT-assisted sample process which allows for complete decrosslinking and trypsin digestion, with comprehensive and accurate protein quantification using the SWATH method (referred to as the FFPE-LMD-PCT-SWATH method), to reveal the pathological molecular mechanisms in ADNC +/CAA + capillaries. The protein expression levels of 17 proteins in ADNC +/CAA +/H donors were significantly different from those in ADNC +/CAA − and ADNC −/CAA − donors (BH-adjusted p < 0.05) (Table 2).

**Fig. 5:**
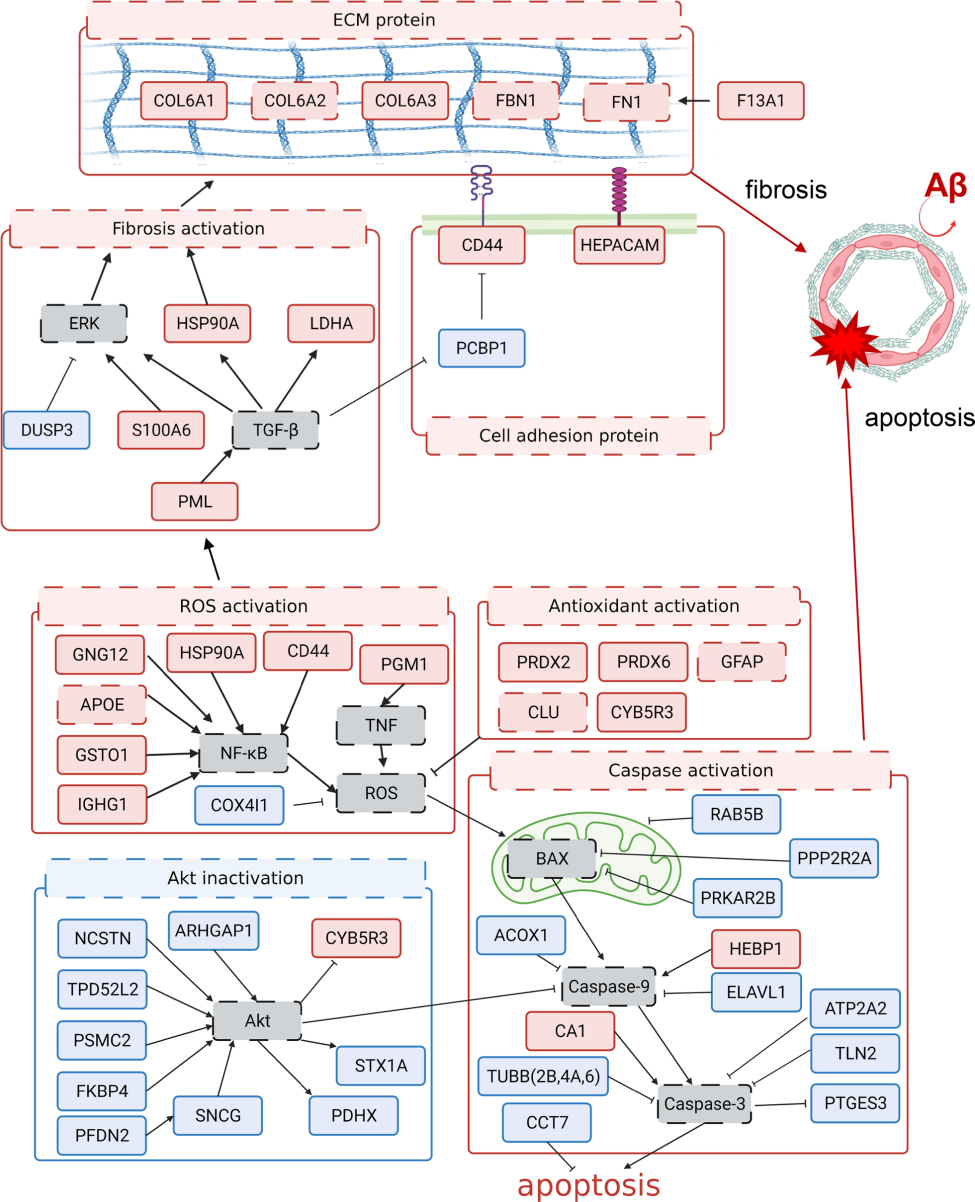
Hypothetical molecular mechanisms in cerebral cortical vessels of CAA patients. Based on the results in the present study, we illustrate the features of pathological molecular mechanisms that occur in cerebral cortical vessels of CAA patients. Red, proteins showing either significant upregulation in ADNC +/CAA +/H donors or significant positive correlations with Aβ or COL6A2. Blue, proteins showing either significant downregulation in ADNC +/CAA +/H donors or significant negative correlations with Aβ or COL6A2. Gray, proteins which were not quantified in the present study. The proteins whose significant change in protein expression in CAA capillary was clarified for the first time in the present study were surrounded with solid line. The proteins whose change in protein expression in CAA capillary or CAA model has been already reported were surrounded with broken line. The mutual relationship of individual molecules was based on our literature search. The present study revealed that a variety of molecules in the fibrosis pathway including ECM, adhesion and TGF-β signal proteins were activated. Furthermore, the activation of oxidative stress-related molecules and the inactivation of AKT signalling molecules are also shown, and these would have activated the apoptosis pathway including caspase signal. The activation of fibrosis and apoptosis leads to the suppression of the Aβ clearance and the destruction of cerebral vessels. Based on literature search, HSP90A, CD44, and CA1 are considered as therapeutic targets of taxifolin, verbascoside, and acetazolamide, respectively. The upregulation of these three proteins in CAA capillaries were clarified for the first time in the present study. The molecular mechanisms illustrated here would be useful for better understanding of the CAA pathology and for promoting the discovery and development of drugs and biomarkers for CAA

More proteins associated with CAA pathology in cerebral capillaries were found in the correlation analysis with typical CAA markers (Aβ or COL6A2). Among the proteins that were quantified, there were 56 proteins that met the following two conditions: (1) proteins that showed more than a 1.5-fold difference in the average expression levels between six ADNC +/CAA + and five ADNC −/CAA − donors for the collected capillaries, and (2) proteins that are significantly (p < 0.05) correlated with Aβ or COL6A2 in 11 donors (6 ADNC +/CAA + and 5 ADNC −/CAA − donors) based on Spearman’s rank correlation analysis. Only 13 of these proteins was correlated significantly (p < 0.05) with Aβ or COL6A2 in the correlation analysis using the data from 10 donors (ADNC +/CAA − and ADNC −/CAA − groups) in the collected capillaries. This suggests that over 70% of the 56 proteins are ADNC +/CAA + specific pathogenic proteins. In the parenchyma, only 3 proteins were significantly correlated with Aβ or COL6A2 in the correlation analysis using the data from 11 donors (ADNC +/CAA + and ADNC −/CAA − groups), suggesting that more than 90% of the 56 proteins are vascular-specific pathological changes (Table 3 and Additional file 2: Table S2). Of these 56 proteins, 42 were associated with fibrosis, oxidative stress and apoptosis. Of these proteins, 36 were identified for the first time in this study as proteins whose expression levels change in ADNC +/CAA + pathology. We now propose more detailed molecular mechanisms for the activation of fibrosis, oxidative stress and apoptosis in ADNC +/CAA + vessels based on these findings as illustrated in Fig. 5.

The results of the present study suggest that fibrosis is activated in ADNC +/CAA + vessels. An increased expression of COL6A2, FBN1 and FN1 [8–10], which are components of the ECM, and activation of TGF-β and ERK, which activate fibrosis by promoting ECM synthesis, have been reported in cerebral blood vessels of ADNC +/CAA + patients [11]. In this study, we show, for the first time, that the expression of COL6A1, COL6A3, and F13A1 (Fig. 2), which contributes to the stabilization of FN1, are increased [12]. We also found an increased expression of S100A6 [13], which activates ERK, and a decreased expression of DUSP3 [14], which represses ERK. In addition, we showed that the protein expressions of PML [15], HSP90A [16] and LDHA [17], which are up- or down-stream of TGF-β signalling, are upregulated. Taxifolin is a compound that is currently attracting attention as a potential treatment for ADNC +/CAA +. In fact, it has been reported to exert multifaceted effects, that include inhibiting the aggregation of Aβ oligomers, stimulating their clearance from cerebral blood vessels, and reducing Aβ production via the inhibition of Nf-κB signalling [18, 19]. However, not all of its mechanisms of action in ADNC +/CAA + are known. It is thought that the clearance of Aβ from blood vessels is impaired by fibrosis [20]. It is therefore possible that inhibiting fibrosis may improve the clearance of Aβ [9]. Interestingly, taxifolin has been reported to inhibit the activation of HSP90A [21]. As illustrated in Fig. 5, the inhibition of HSP90A suppresses the activation of both fibrosis and Nf-κB signalling [22]. Therefore, taxifolin may inhibit fibrosis and Nf-κB signalling by targeting HSP90A in ADNC +/CAA +, thereby enhancing Aβ clearance and inhibiting its production.

Verbascoside is a compound that has also attracted attention as a therapeutic agent for ADNC +/CAA +. It has been reported to protect cells from oxidative stress by reducing ROS levels and to improve the clearance of Aβ from cerebral blood vessels when administered to CAA model mice [23, 24]. However, the details of the molecular mechanism of action with regard to protecting cells from oxidative stress and improving clearance remain unclear. In this study, increased expressions of CD44 [25] and HEPACAM [26], which promote fibrosis by promoting cell-ECM adhesion, and a decreased expression of PCBP1 [27], which inhibits CD44, were observed in cortical vessels from ADNC +/CAA + patients for the first time (Fig. 2). Interestingly, verbascoside has been reported to inhibit the activity of CD44 in glioblastoma cell lines [28]. These finding suggest, therefore, that verbascoside inhibits fibrosis by inhibiting CD44. The inhibition of fibrosis, as described above, improves the clearance of Aβ. It has also been reported that the inhibition of CD44 suppresses Nf-kB signalling, leading to the suppression of oxidative stress [29]. Therefore, verbascoside may also target CD44 in ADNC +/CAA + endothelial cells, thereby inhibiting fibrosis and Nf-kB signalling, improving Aβ clearance and protecting cells from oxidative stress (Fig. 5).

The expression of APOE is increased in ADNC +/CAA +, and Nf-κB signaling is activated to promote ROS production [8, 30]. Increased expression of GNG12 [31], GSTO1 [32], and IGHG1 [33], which activate Nf-κB signaling, and the decreased expression of COX4I1 which inhibits ROS production [34], are demonstrated herein for the first time in the cerebral blood vessels of ADNC +/CAA + patients (Figs. 3 and 5). It has also been reported that the level of LPS, which activates Nf-κB signalling and increases ROS production, is upregulated by three-fold in the blood of ADNC +/CAA + patients compared to normal blood and the LPS co-localizes with Aβ in cerebral blood vessels [35, 36]. Interestingly, the knockdown of GSTO1 has been reported to dramatically reduce the LPS-stimulated production of ROS [37]. Therefore, the targeted inhibition of GSTO1 may have the potential for the treatment of ADNC +/CAA + oxidative stress-induced damage (Fig. 5).

An increased expression of antioxidant proteins, including CLU and GFAP, has also been reported in ADNC +/CAA + vessels. These proteins act in a cytoprotective manner against oxidative stress when increased [8, 38–40]. In the present study, we found, for the first time, that the expressions of the antioxidant proteins PRDX2 and PRDX6 are increased [41], which are up-regulated in response to increased oxidative stress, and CYB5R3 [42], which is activated upon the inactivation of AKT signaling (Figs. 3 and 5), in ADNC +/CAA + vessels. It is possible that the increased expression of these proteins function to protect the cells from oxidative stress (Fig. 5).

For AKT signalling (Fig. 5), we found the decreased expressions of NCSTN [43], TPD52L2 [44], PSMC2 [45], FKBP4 [46] and ARHGAP1 [47] which activate AKT signalling (Fig. 4). The decreased expression of PDHX [48] and STX1A [49] which are activated by AKT signaling, and the increased expression of CYB5R3 [42] which is activated by inactivation of AKT signaling were also shown in ADNC +/CAA + vessels (Fig. 4). These results suggest that AKT signalling is suppressed in ADNC +/CAA + vessels (Fig. 5). In fact, an in vitro study reported that Aβ exposure inactivates AKT signalling in endothelial cells [50].

It has been reported that BAX, caspase 3 and caspase 9 are activated in ADNC +/CAA + and promote apoptosis (Fig. 5) [1, 51, 52]. In this study, we found, for the first time, that the expression of RAB5B [53] which promotes the degradation of damaged mitochondria was decreased, the expression of PPP2R2A [54] which represses BAX, and the expression of PRKAR2B [55, 56] which represses BAX via the pentose phosphate pathway were decreased in the cerebral blood vessels of ADNC +/CAA + patients (Fig. 4). In addition, the increased expression of HEBP1 [57] which activates caspase 9, and the decreased expression of ACOX1 [58] which represses caspase 9, are reported (Fig. 4). The decreased expression of TUBB2B, TUBB4A, TUBB6 [59] and ATP2A2 [60] which repress the action of caspase 3, and the decreased expression of PTGES3 [61] which is repressed by the action of caspase 3, and increased expression of carbonic anhydrase 1 (CA1) [62] which activates caspase 3, are also shown for the first time (Fig. 4). Acetazolamide is a CA1 inhibitor that is used in the treatment of epilepsy and as a diuretic, and has been reported to inhibit apoptosis in human brain vascular endothelial cells that had been exposed to Aβ [63]. Although the inhibition of CA may inhibit apoptosis, the issue of whether CA is actually activated in the cerebral vessels of ADNC +/CAA + patients remains unclear. The present study revealed an increased expression of CA1 in ADNC +/CAA + vessels. These results suggest that acetazolamide inhibits apoptosis by targeting the induced expression of CA1 (Fig. 5).

In the present study, vessels were sampled by LMD and, in addition to the endothelium, they were also found to contain neurovascular units (NVU) such as astrocytes and pericytes; GFAP and CD44 are astrocyte markers and their expression was elevated in ADNC +/CAA + vessels, suggesting astrocyte activation. The ability to produce collagen is greater in pericytes, suggesting that the increase in COL6A1, COL6A2 and COL6A3 in the ADNC +/CAA + vessels is due to pericyte activation. However, many of the molecules shown in Fig. 5 are types of proteins that are expressed by both endothelial cells, astrocytes and pericytes. This makes it difficult to distinguish between different cellular molecular mechanisms based on the results of this study alone. In the future, to elucidate how CAA affects NVU as a whole, cell-distinct proteomic analyses in NVUs will be needed.

Measuring a large number of readouts in a proteomic analysis increases the chance of identifying statistically significant changes that may not be true. A Benjamini-Hochberg (BH) correction may help but this does not completely address this issue. Furthermore, when comparing the concentration of two entities from the same samples that are undergoing the same processing steps, the probability of detecting a positive association is high. Therefore, inverse associations may be more meaningful because there are devoid of such methodological bias. In any case, the certainty of the results reported in this study may not be sufficient due to the small number of donors. The criterion for the p-value was set at 0.05, but 0.01 might be more reasonable for a correlation analysis. In the future, additional quantitative analyses using different cohorts with larger number of donor samples will be needed. This would allow the results obtained in this study to be more likely to be valid. In addition, confirmatory experiments using other methods would also be desirable.

It is interesting to compare our proteomic data with data reported in previous CAA proteomics studies. In two studies, the brain vessels were isolated from CAA frozen brains and used for proteomic analysis, but the COL6A family, which is directly involved in fibrosis, a typical CAA pathology, was not detected or was not increased in CAA vessels [10, 64]. CAA vessels are very fragile and the vascular structure is disrupted during the process of vessel isolation. Also, not all of the vessels in the brain tissue used necessarily exhibited CAA pathology (some normal vessels could be included in the samples). Therefore, the proteomic data reported in these studies may not sufficiently reflect the true pathological molecular mechanisms in the CAA vasculature. In 2018, another proteomic analysis of CAA brains was performed using LMD, but the vessels were not enriched and basically the brain parenchyma regions, including vessels, were cut out for proteomic experiment [8]. Although an increase in the COL6A family was found, the pathological molecular mechanisms in blood vessels were not adequately captured because the majority of the cells were parenchymal cells. We addressed these issues and were able to properly quantify the pathological molecular mechanisms of CAA vessels. Since we were able to capture the changes in protein expression that is associated with fibrosis, oxidative stress and cell death that occur in CAA (Fig. 5), we conclude that we were able to properly elucidate the pathological molecular mechanisms associated with CAA vessels.

FFPE sections are very widely used in clinical practice and a huge number of specimens have been collected and stored, with the relevant accompanying clinical information, in medical facilities worldwide. Traditionally, formalin cross-linking has been a major barrier to conducting proteomics experiments. However, we were able to develop a PCT-based sample process technique that allows the complete de-crosslinking and trypsin digestion of a sample, and a highly accurate SWATH-based comprehensive quantification method. This enabled us to properly quantify the true pathological changes that occur in vivo by using such FFPE sections [3]. It is expected that this method will be used in the future to elucidate the pathological molecular mechanisms associated with various human diseases, not only CAA, and to identify specific biomarkers.

## Conclusions

The combination of LMD-based isolation of vessels from FFPE sections, PCT-assisted sample processing and SWATH analysis (FFPE-LMD-PCT-SWATH method) revealed for the first time the changes in the expression of many proteins that are involved in fibrosis, ROS production and cell death in ADNC +/CAA + vessels. ADNC +/CAA + showed an increased expression of HSP90A and CD44, which are therapeutic targets of taxifolin and verbascoside. In addition, for the first time, we show the increased expression of CA1, a therapeutic target of acetazolamide that inhibits ADNC +/CAA + cell death. It is expected that the molecular mechanisms clarified in the present study (Fig. 5) will be useful for developing a better understanding of the pathology of CAA and for promoting the discovery and development of drugs and biomarkers for CAA.

## Supplementary Information

**Additional file 1: Figure S1.** CAA-specific changes in expression levels of the proteins other than fibrosis, oxidative stress, AKT and apoptosis signals in the collected capillaries. **Figure S2.** Fibrosis-associated molecules shown with different graphs from Fig. 2. **Figure S3.** Oxidative stress-associated molecules shown with different graphs from Fig. 3. **Figure S4.** Apoptosis-associated molecules shown with different graphs from Fig. 4. **Figure S5.** Unclassified other proteins shown with different graphs from Additional file 1: Figure S1.

**Additional file 2: Table S1.** Raw SWATH data for the isolated capillary and parenchyma. **Table S2.** Spearman’s rank correlation with COL6A2 to extract CAA-specific and vascular-specific pathological changes.

## Data Availability

The data that support the findings of this study are available from the corresponding author (Yasuo Uchida) upon reasonable request.
