# Supplementary material for: Proteomics-based investigation of cerebrovascular molecular mechanisms in cerebral amyloid angiopathy by the FFPE-LMD-PCT-SWATH method
Source: Fluids Barriers CNS. 2022 Jul 1;19:56. doi: 10.1186/s12987-022-00351-x (PMC9250250; doi:10.1186/s12987-022-00351-x)
Supplement: Supplementary file 1 — Additional file 1: Figure S1. CAA-specific changes in expression levels of the proteins other than fibrosis, oxidative stress, AKT and apoptosis signals in the collected capillaries. Figure S2. Fibrosis-associated molecules shown with different graphs from Fig. 2. Figure S3. Oxidative stress-associated molecules shown with different graphs from Fig. 3. Figure S4. Apoptosis-associated molecules shown with different graphs from Fig. 4. Figure S5. Unclassified other proteins shown with different graphs from Additional file 1: Figure S1. [file 12987_2022_351_MOESM1_ESM.pdf]

Supplementary materials

Title:

Proteomics-based investigation of cerebrovascular molecular mechanisms in cerebral amyloid angiopathy by the FFPE-LMD-PCT-SWATH method

Takumi Handa,[1] Hayate Sasaki,[1] Masaki Takao,[2,3] Mitsutoshi Tano,[2] and Yasuo Uchida[1]

[1]Graduate School of Pharmaceutical Sciences, Tohoku University, Sendai, Japan

[2]Department of Neurology and Brain Bank, Mihara Memorial Hospital, Japan

[3]Department of Clinical Laboratory, National Center of Neurology and Psychiatry, National Center Hospital, Japan

Corresponding author: Yasuo Uchida, Ph.D.

Division of Membrane Transport and Drug Targeting, Graduate School of Pharmaceutical Sciences, Tohoku University, 6-3 Aoba, Aramaki, Aoba-ku, Sendai, 980-8578, Japan.

Voice: +81-22-795-6832; FAX: +81-22-795-6886; E-mail: yasuo.uchida.c8@tohoku.ac.jp

List of supplementary materials

Figures S1 to S5

Tables S1 and S2 (Uploaded as Excel file)

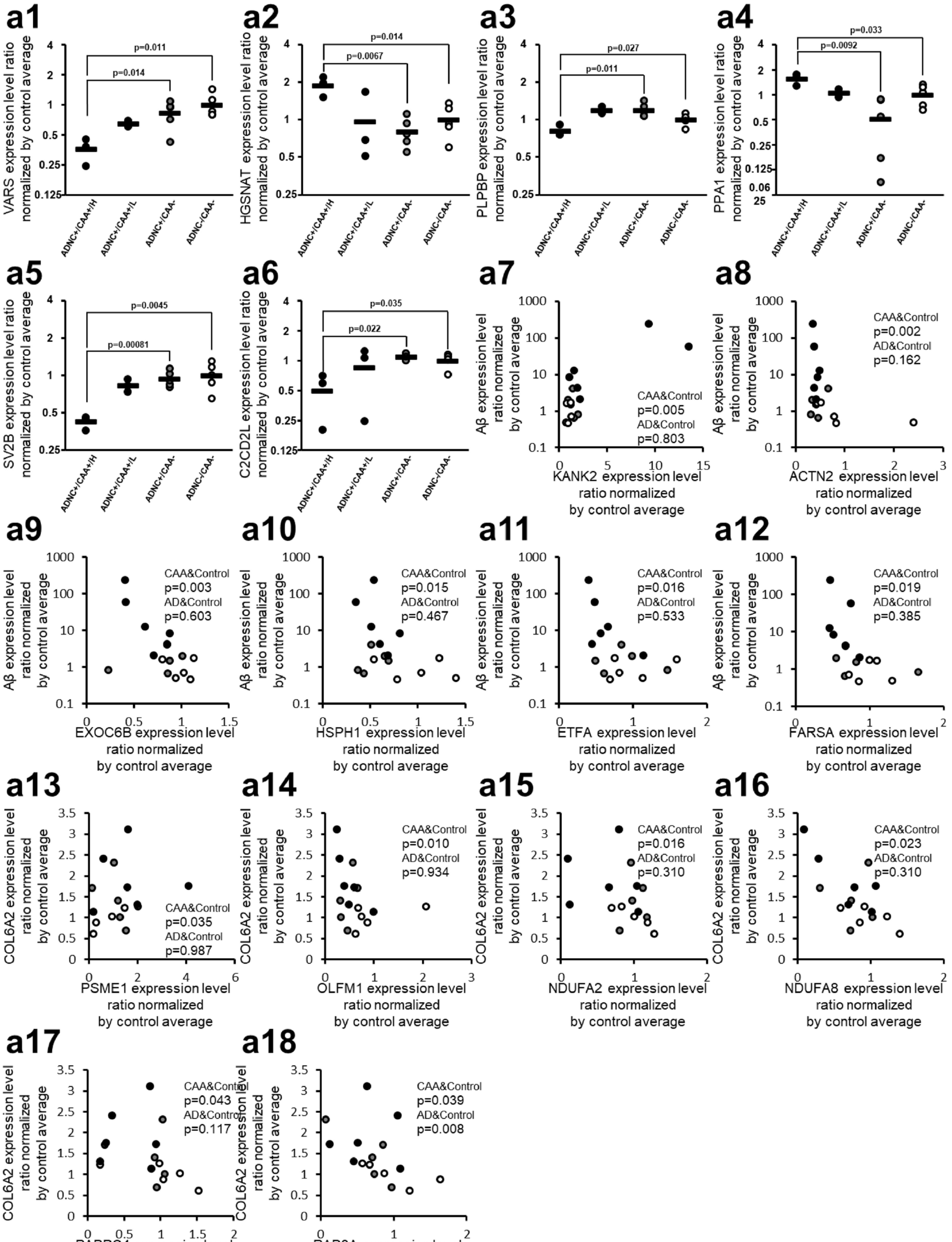

Figure S1 CAA-specific changes in expression levels of the proteins other than fibrosis, oxidative stress, AKT and apoptosis signals in the collected capillaries

For unclassified other proteins listed in Table 2, the graphs showing their intergroup comparisons are preferentially presented in this figure. For unclassified other proteins not listed in Table 2 but listed in Table 3 or Table S2, the correlation graphs with Aβ are shown in this figure if the protein showed a significant correlation both with Aβ (Table 3) and COL6A2 (Table S2). The data for protein expression levels for individual donors (normalized by average of protein expression levels in ADNC-/CAA- capillaries) were taken from Table S1. Black plot, ADNC+/CAA+ (CAA); Gray plot, ADNC+/CAA- (AD); White plot, ADNC-/CAA- (Control). For the ADNC+/CAA+ group, the three donors with highly abundant Aβ in capillaries (donors 1, 2 and 3) were classified as "ADNC+/CAA+/H", and the other three donors (donors 4, 5 and 6) were classified as "ADNC+/CAA+/L". The p-values were taken from Table 2, Table 3 or Table S2. For the correlation graphs, the p-values of "CAA&Control" and "AD&Control" represent the ones in Spearman's rank correlation using 11 donors (ADNC+/CAA+ and ADNC-/CAA-) and 10 donors (ADNC+/CAA- and ADNC-/CAA-), respectively.

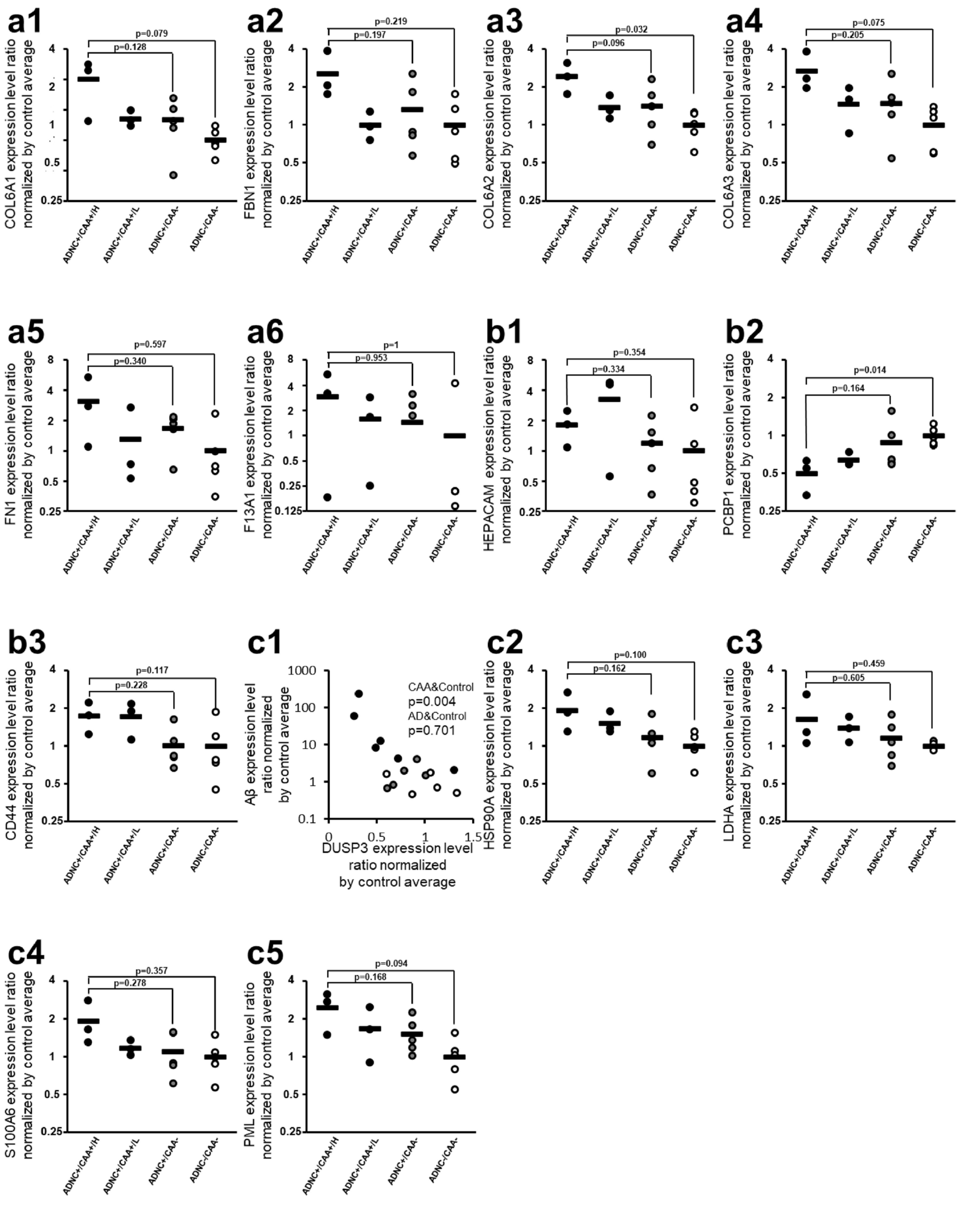

Figure S2 Fibrosis-associated molecules shown with different graphs from Figure 2

The same proteins as Figure 2 are presented. For the proteins which are presented with the graphs of intergroup comparisons in Figure 2, the correlation graphs with Aβ or COL6A2 are shown in this figure. For the proteins which are presented with the correlation graphs in Figure 2, the graphs of intergroup comparisons are presented in this figure. (a) ECM-related proteins, (b) Cell-ECM adhesion-related proteins, (c) TGF-β signal-related proteins. The data for protein expression levels for individual donors (normalized by average of protein expression levels in ADNC-/CAA- capillaries) were taken from Table S1. Black plot, ADNC+/CAA+ (CAA); Gray plot, ADNC+/CAA- (AD); White plot, ADNC-/CAA- (Control). For the ADNC+/CAA+ group, the three donors with highly abundant Aβ in capillaries (donors 1, 2 and 3) were classified as "ADNC+/CAA+/H", and the other three donors (donors 4, 5 and 6) were classified as "ADNC+/CAA+/L". The p-values were taken from Table S1. For the correlation graphs, the p-values of “CAA&Control” and “AD&Control” represent the ones in Spearman's rank correlation using 11 donors (ADNC+/CAA+ and ADNC-/CAA-) and 10 donors (ADNC+/CAA- and ADNC-/CAA-), respectively.

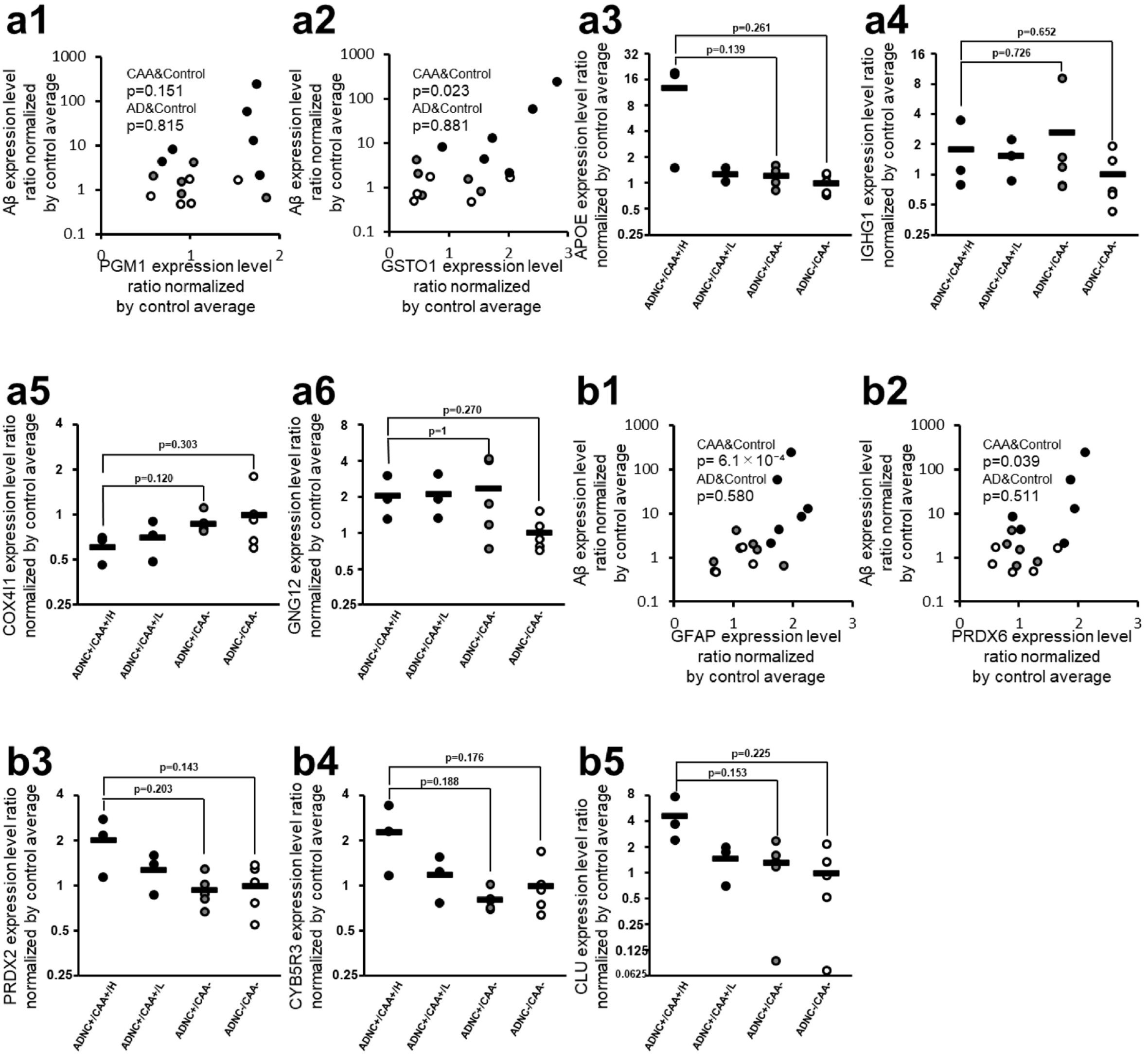

Figure S3 Oxidative stress-associated molecules shown with different graphs from Figure 3

The same proteins as Figure 3 are presented. For the proteins which are presented with the graphs of intergroup comparisons in Figure 3, the correlation graphs with Aβ or COL6A2 are shown in this figure. For the proteins which are presented with the correlation graphs in Figure 3, the graphs of intergroup comparisons are presented in this figure. (a) ROS-related proteins, and (b) Antioxidant-related proteins. The data for protein expression levels for individual donors (normalized by average of protein expression levels in ADNC-/CAA- capillaries) were taken from Table S1. Black plot, ADNC+/CAA+ (CAA); Gray plot, ADNC+/CAA- (AD); White plot, ADNC-/CAA- (Control). For the ADNC+/CAA+ group, the three donors with highly abundant Aβ in capillaries (donors 1, 2 and 3) were classified as "ADNC+/CAA+/H", and the other three donors (donors 4, 5 and 6) were classified as "ADNC+/CAA+/L". The p-values were taken from Table S1. For the correlation graphs, the p-values of “CAA&Control” and “AD&Control” represent the ones in Spearman's rank correlation using 11 donors (ADNC+/CAA+ and ADNC-/CAA-) and 10 donors (ADNC+/CAA- and ADNC-/CAA-), respectively.

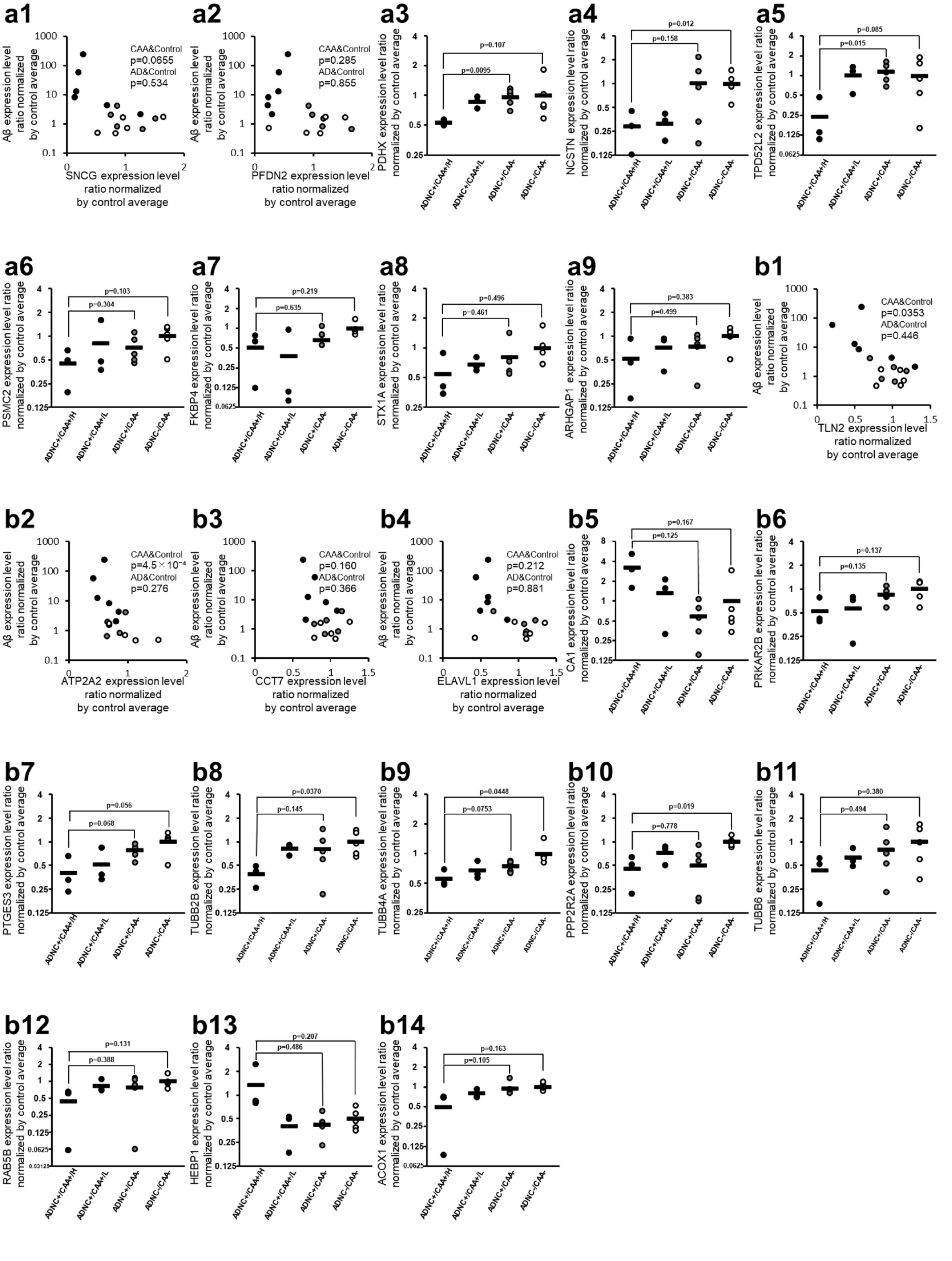

Figure S4 Apoptosis-associated molecules shown with different graphs from Figure 4

The same proteins as Figure 4 are presented. For the proteins which are presented with the graphs of intergroup comparisons in Figure 4, the correlation graphs with Aβ or COL6A2 are shown in this figure. For the proteins which are presented with the correlation graphs in Figure 4, the graphs of intergroup comparisons are presented in this figure. (a) AKT signal-related proteins, and (b) Caspase signal-related proteins. The data for protein expression levels for individual donors (normalized by average of protein expression levels in ADNC-/CAA- capillaries) were taken from Table S1. Black plot, ADNC+/CAA+ (CAA); Gray plot, ADNC+/CAA- (AD); White plot, ADNC-/CAA- (Control). For the ADNC+/CAA+ group, the three donors with highly abundant Aβ in capillaries (donors 1, 2 and 3) were classified as "ADNC+/CAA+/H", and the other three donors (donors 4, 5 and 6) were classified as "ADNC+/CAA+/L". The p-values were taken from Table S1. For the correlation graphs, the p-values of "CAA&Control" and "AD&Control" represent the ones in Spearman's rank correlation using 11 donors (ADNC+/CAA+ and ADNC-/CAA-) and 10 donors (ADNC+/CAA- and ADNC-/CAA-), respectively.

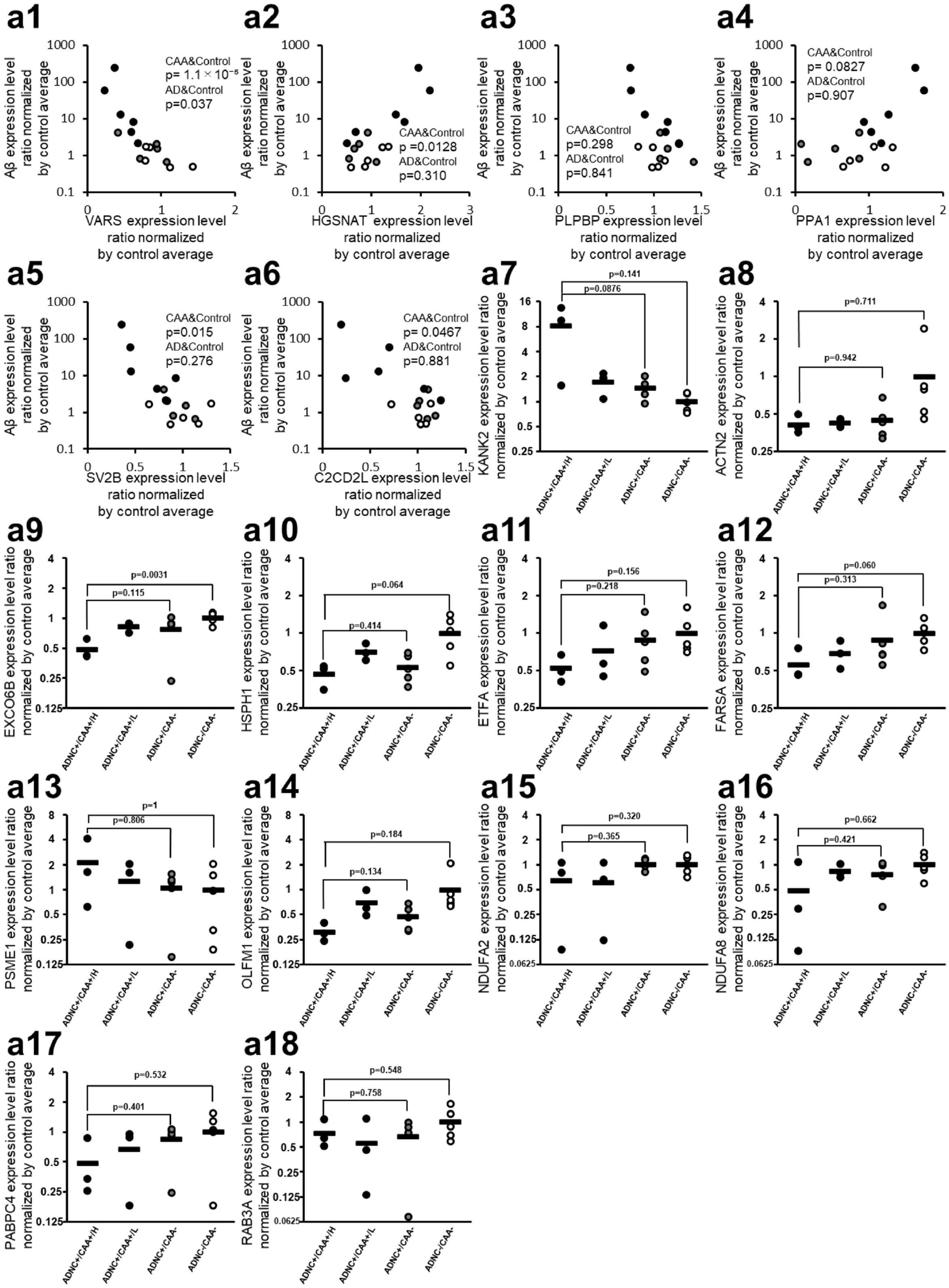

Figure S5 Unclassified other proteins shown with different graphs from Figure S1

The same proteins as Figure S1 are presented. For the proteins which are presented with the graphs of intergroup comparisons in Figure S1, the correlation graphs with Aβ or COL6A2 are shown in this figure. For the proteins which are presented with the correlation graphs in Figure S1, the graphs of intergroup comparisons are presented in this figure. The data for protein expression levels for individual donors (normalized by average of protein expression levels in ADNC-/CAA- capillaries) were taken from Table S1. Black plot, ADNC+/CAA+ (CAA); Gray plot, ADNC+/CAA- (AD); White plot, ADNC-/CAA- (Control). For the ADNC+/CAA+ group, the three donors with highly abundant Aβ in capillaries (donors 1, 2 and 3) were classified as "ADNC+/CAA+/H", and the other three donors (donors 4, 5 and 6) were classified as "ADNC+/CAA+/L". The p-values were taken from Table S1. For the correlation graphs, the p-values of "CAA&Control" and "AD&Control" represent the ones in Spearman's rank correlation using 11 donors (ADNC+/CAA+ and ADNC-/CAA-) and 10 donors (ADNC+/CAA- and ADNC-/CAA-), respectively.
